# Hidden fungal diversity from the Neotropics: *Geastrum hirsutum*, *G*. *schweinitzii* (Basidiomycota, Geastrales) and their allies

**DOI:** 10.1371/journal.pone.0211388

**Published:** 2019-02-06

**Authors:** Thiago Accioly, Julieth O. Sousa, Pierre-Arthur Moreau, Christophe Lécuru, Bianca D. B. Silva, Mélanie Roy, Monique Gardes, Iuri G. Baseia, María P. Martín

**Affiliations:** 1 Programa de Pós-Graduação em Sistemática e Evolução, Universidade Federal do Rio Grande do Norte, Natal, Rio Grande do Norte, Brazil; 2 EA4483 IMPECS, UFR Pharmacie, Université de Lille, Lille, France; 3 Herbarium LIP, UFR Pharmacie, Université de Lille, Lille, France; 4 Departamento de Botânica, Instituto de Biologia, Universidade Federal da Bahia, Ondina, Salvador, Bahia, Brazil; 5 Laboratoire UMR5174 Evolution et Diversité Biologique (EDB), Université Toulouse 3 Paul Sabatier, Toulouse, France; 6 Departamento de Botânica e Zoologia, Universidade Federal do Rio Grande do Norte, Natal, Rio Grande do Norte, Brazil; 7 Departamento de Micología, Real Jardín Botánico-CSIC, Madrid, Spain; Universita degli Studi di Pisa, ITALY

## Abstract

Taxonomy of *Geastrum* species in the neotropics has been subject to divergent opinions among specialists. In our study, type collections were reassessed and compared with recent collections in order to delimit species in *Geastrum*, sect. *Myceliostroma*, subsect. *Epigaea*. A thorough review of morphologic features combined with barcode and phylogenetic analyses (ITS and LSU nrDNA) revealed six new species (*G*. *neoamericanum*, *G*. *rubellum*, *G*. *brunneocapillatum*, *G*. *baculicrystallum*, *G*. *rubropusillum* and *G*. *courtecuissei*). In additon, the presence of hairs on the exoperidium, a commonly used feature to diagnose *Geastrum* species, proved to be ineffective because it is a derived character within subsect. *Epigaea*.

## Introduction

The Neotropical biogeographic realm, or Neotropics, comprises Central America, most of South America (except Patagonia), and the southern portion of North America. It is considered the most diverse region for well-studied terrestrial taxa, mainly animals (amphibians, reptiles, birds and mammals) and plants (Angiosperms) [1,2,3,4,5]. However, knowledge regarding neotropical fungi is still insufficient. Indeed, the neotropical region represents a priority area for taxonomic studies since it encompasses megadiverse countries (Brazil, Colombia, Costa Rica, Ecuador, Mexico, Peru, Venezuela) with hotspot areas, such as the Atlantic Rainforest, Cerrado and Caribbean Islands; and tropical ecosystems where many potentially new taxa are threatened by human impacts [6,7,8,9].

*Geastrum* Pers., is a genus of gasteroid fungi in which the outer layers of the fruitbodies (basidiomata) open in a stellate pattern once the spores are mature, which makes them popularly known as *earthstars*. Moreover, *Geastrum* is one of the most diverse genera of gasteroid fungi, with 100–120 species around the world [10]. Although intensive inventories of gasteroid fungi in Brazil began only in the last two decades, already 56 *Geastrum* species have been reported or described from this country, which accounts for approximately half of the global *Geastrum* diversity. In recent years, at least one new species per year has been described from Brazil, including *Geastrum ishikawae* Accioly et al.; *G*. *pusillipilosum* J.O. Sousa et al.; *G*. *verrucoramulosum* T.S. Cabral, J.O. Sousa & Baseia; *G*. *caririense* R.J. Ferreira et al.; *G*. *magnosporum* J.O. Sousa et al. [11,12,13,14,15].

However, a great diversity remains hidden, especially in poorly sampled areas, as well as within species that have been considered of wide distribution, such as *Geastrum triplex* Jungh. [16]. Based on molecular data (ITS and LSU nrDNA loci; as well as a fragment of the *atp*-6 gene), Kasuya et al. [16] reported different groups according to their geographical origin, confirming that the epithet *G*. *triplex* represents, in fact, a complex of cryptic or semi-cryptic species [17,18,19,20,21] awaiting description.

In another recent study about species complexes in sect. *Geastrum*, Zamora et al. [22] discovered seven new species through integrative taxonomy by analysing not only molecular data, but also chemical and ultra-structural features usually not studied in the group. However, these studies reported mainly efforts on temperate or sub-tropical species, without much attention to tropical and neotropical regions, where greater diversity is expected [7,23,24].

Subiculose *Geastrum* species are generally found in tropical habitats and are encompassed by section *Myceliostroma* [10]. The subiculum is a macroscopic, whitish layer, composed of thin, interlaced, sinuous hyphae [25]. This type of mycelium occurs mainly in lignicolous species, but it was also recorded in species growing on soil [26]. The most widespread neotropical species with this feature are *Geastrum schweinitzii* (Berk. & M.A. Curtis) Zeller and *G*. *hirsutum* Baseia & Calonge.

Both *G*. *schweinitzii* and *G*. *hirsutum* are included in subsect. *Epigaea* Dissing & M. Lange within sect. *Myceliostroma* (P. Henn.) P. Ponce de León, and there has been an on-going controversy about their identities. In 1989, Ponce de León synonymized ten epithets under the name *G*. *schweinitzii*, including *G*. *mirabile* Mont., *G subiculosum* Cooke & Massee, and *G*. *trichiferum* Rick; and considered a stipitate variety: *Geastrum schweinitzii* var. *stipitatum* (Solms) P. Ponce de León [27]. The species concept of Ponce de León has been widely applied by others since then.

When the epithet *G*. *trichiferum* (= *Geaster trichifer*) was revived by Trierveiler-Pereira & Silveira [28], *G*. *hirsutum* was synonymized into it, mainly based on its hirsute exoperidium. However, the identity of these hairy species is controversial. [29,30].

This study aimed to enhance the knowledge about neotropical *Geastrum* species richness and taxonomy by investigating potential morphospecies complexes within sect. *Myceliostroma*, subsect *Epigaea*.

## Materials and methods

### Morphological studies

Macro and micro morphological analyses were performed with fresh and mainly with dry basidiomata from André Maurício Vieira de Carvalho Herbarium, Cocoa Research Center (CEPEC, Itabuna-BA, Brazil); Fungal Collection of the Federal University of Rio Grande do Norte (UFRN–Fungos, Natal-RN, Brazil); Herbarium Anchieta (PACA, Porto Alegre-RS, Brazil); Kew Fungarium of the Royal Botanic Garden (K(M), London, England); National Institute of Amazonian Research (INPA, Manaus-AM, Brazil); U.S. National Fungus Collections (BPI, Beltsville-MD, USA). The descriptions were based on specific literature [10,25,31,32,33]. Comparative analyses were done including the following type collections: *Geastrum hirsutum*, Brazil–Pernambuco, Recife, Reserva Ecológica do Gurjaú, 12 Jul. 2003, leg. I.G. Baseia (Ma-Fungi 67886 paratype); *Geastrum pleosporum*, Cameroon, Centre Province, Department of Nyong & So’o, in the Mbalmayo forest reserve, 47 km South east of Yaounde, 16 Oct. 2002, leg. C. Douanla–Meli (MA–Fungi 56971 isotype); *Geastrum pusillipilosum*, Brazil, Paraíba, Mamanguape, REBIO Guaribas, SEMA II, 26 Jun.2014, leg. J.O. Sousa JM100 (UFRN–Fungos 2315 holotype, ITS sequence GenBank KX761175); *Geastrum trichiferum* PACA 15970, BPI 706088 (Rick’s original collection), and BPI 706086; and *Geastrum schweinitzii*, suriname, (K(M) 180187 type, K(M) 180187 isotype).

Colour descriptions were based on [34,35]. Sample observations and macro morphological image capturing were done using a stereomicroscope Nikon H600L coupled with a Nikon DS–Ri camera. Micro morphological studies were carried out using a Nikon Eclipse Ni light microscope (LM) coupled with a Nikon DS–Ri camera, and scanning electron microscope (SEM) analysis was done under a Shimadzu SSX–550. For light microscopy of basidiospores, eucapillitium, rhizomorphs and exoperidial hyphae, the samples were mounted in 5% KOH (w/v). Also, samples were mounted in Congo Red to observe the basidia; and Melzer’s reagent was used to test the exoperidium, subiculum and rhizomorph hyphae. Preparation of the material examined under SEM followed Silva et al. [36]. At least thirty randomly selected basidiospores were measured using LM at 1000× magnification, including surface ornamentation; and the height of the ornamentation was also measured. Basidiospore abbreviations follow [37]: n = number of randomly measured basidiospores; x = mean ± standard deviation of basidiospore diameter and height (including ornamentation); Q_m_ = mean height/width quotient. Geographic distributions of delimited taxa followed Biogeographic Realms, Biomes and Ecoregions proposed by Dinerstein et al. [5].

### Molecular analyses

For UFRN–Fungos, INPA-Fungos and CEPEC samples, the extractions of DNA were performed utilizing 10 mg of gleba from dry basidiomata, preferably mature gleba. For the DNA isolation, DNeasyTMPlant Mini Kit (Qiagen, Valencia, CA) was used following manufacturer’s instructions; except that the incubation in the lysis buffer was done at 55–60 °C overnight. For all other samples, fungal DNA was extracted from fragments of dried fruitbodies by using the Wizard Genomic Purification kit (Promega, Charbonnière les Bains, France) according to the manufacturer’s recommendations, and the final pellet resuspended in 40 μl of sterile water. Internal Transcribed Spacer (ITS) region of the nuclear ribosomal gene, including the 5.8S subunit (ITS nrDNA), and Large Subunit region of nuclear ribosomal DNA (LSU nrDNA) were the loci selected for molecular analyses. DNA amplification, purification and sequencing protocols are deposited in protocols.io (dx.doi.org/10.17504/protocols.io.wpdfdi6).

Sequences obtained in this study were submitted to Genbank under the accession numbers indicated in Table 1. The newly-generated ITS and LSU sequences, and homologous sequences retrieved from EMBL/GenBank/DDBJ databases, mainly from [10,12,16,38], were separately aligned in MEGA7: Molecular Evolutionary Genetics Analysis version 7.0 [39]. Alignment gaps were marked with “–” and unresolved nucleotide positions were indicated with “N”. *Geastrum velutinum* was included as outgroup since this species is in section *Myceliostroma*, subsection *Velutina* J.C. Zamora, sister clade of subsect. *Epigaea* [10].

**Table 1:** Specimens and sequences included in this study. Clades as indicated from the bottom-up in Fig 1. Accession numbers in bold are from newly generated sequences.

Clade—Species	Fungarium Number	Country	Genbank Acc. N° ITS	Genbank Acc. N° LSU
Clade I—*G*. *schweinitzii*	K(M)180188 Type	Suriname	-	-
	K(M) 180187 Isotype	Suriname	**MH635016**	**-**
	INPA 143435	Brazil	**MH635017**	-
Clade II—*G*. *mirabile*	TNS 36758	Japan, Bonin Island	JN845108	JN845226
	TNS 36761	Japan, Bonin Island	JN845109	JN845227
Clade IIII—*G*. *courtecuissei* sp. nov.	LIP PAM/GUAD 11.04 Paratype	Guadeloupe Island (France)	**MH635002**	-
	LIP FH2004090503 Holotype	Guadeolupe Island (France)	**MH635003**	**MH635033**
	MA–Fungi 83779 (under *G*. *schweinitzii*) Paratype	Argentina	KF988437	KF988567
Clade IV—*G*. *pleosporum*	MA–Fungi 56971 Isotype	Cameroon	KF988416	KF988544
Clade V—*G*. *hirsutum*	LIP MR/GUY 12.171	French Guiana	**MH635004**	-
	LIP RC/GUY 12.086	French Guiana	**MH635005**	-
	INPA 259950	Brazil	**MH634993**	**MH635026**
	MA–Fungi 67886 Paratype	Brazil	**MH538295**	-
Clade VI—*G*. *rubropusillum* sp. nov.	LIP RC/MART 03.015 Holotype	France, Martinique Island	**MH635006**	-
	LIP CL/MART 08.112 Paratype	France, Martinique Island	**MH635007**	**MH635034**
	MA–Fungi 36141 (under *G*. *schweinitzii*) Paratype	Panama	KF988438	KF988568
	UFRN–Fungos 2308 Paratype	Brazil	**MH634994**	**MH635027**
Clade VII—*G*. *pusillipilosum*	MA–Fungi 83780 (under *G*. s*chweinitzii*)	Argentina	KF988439	KF988569
	UFRN–Fungos 2315 Holotype	Brazil	KX761175	KX761176
	UFRN–Fungos 2256 Paratype	Brazil	KX761180	-
	UFRN–Fungos 2759	Brazil	KX761177	KX761178
Clade VIII—*G*. *baculicrystallum* sp. nov.	LIP RC/GUY 08.035 Paratype	French Guiana	**MH635008**	-
	UFRN–Fungos 2835 Holotype	Brazil	**MH634995**	**MH635028**
	UFRN–Fungos 1857 **P**aratype	Brazil	**MH635018**	**MH635035**
Clade IX—*G*. *brunneocapillatum* sp. nov.	UFRN–Fungos 2286 Holotype	Brazil	**MH634996**	**MH635029**
	UFRN–Fungos 2834 Paratype	Brazil	**MH634997**	**MH635030**
	UFRN–Fungos 2851 Paratype	Costa Rica	**MH634998**	-
Clade X—*G*. *minutisporum*	CORD-MLHC 14 Holotype	Argentina	KM260664	-
	CORD-MLHC 15	Argentina	KM260665	-
	CORD-MLHC 16	Argentina	KM260666	-
Clade XI—*G*. *rubellum* sp. nov.	LIP CL/MART 08.067B Paratype	France, Martinique Island	**MH635009**	**MH635035**
	LIP PAM/MART 12.100 Holotype	France, Martinique Island	**MH635010**	**MH635037**
	UFRN–Fungos 1214 (under *G*. *hirsutum*) Paratype	Brazil	KJ127029	JQ683662
	UFRN–Fungos 2844 Paratype	Brazil	**MH634999**	**MH635031**
Clade XII—*G*. *neoamericanum* sp. nov.	LIP CL/GUAD 06.010 Paratype	France, Guadeloupe Island	**MH635011**	-
	LIP CL/GUAD 06.056 Paratype	France, Guadeloupe Island	**MH635012**	-
	LIP CL/MART 04.001 Paratype	France, Martinique Island	**MH635013**	-
	LIP JLC12030103 Paratype	French Guiana	**MH635014**	**MH635038**
	LIP MR/GUY 12.004 Paratype	French Guiana	**MH635015**	-
	UFRN–Fungos 348 Paratype	Brazil	**MH635019**	-
	UFRN–Fungos 2850 Paratype	Brazil	**MH635000**	**MH635032**
	CEPEC 1391 Paratype	Brazil	**MH635020**	-
	UFRN–Fungos 2149 Paratype	Brazil	**MH635021**	**MH635039**
	UFRN–Fungos 2302 Holotype	Brazil	**MH635001**	**MH635040**
	INPA 259949 Paratype	Brazil	**MH635025**	**MH635041**
	UFRN–Fungos 1741 Paratype	Brazil	KJ127030	**MH635042**
	UFRN–Fungos 2655 Paratype	Brazil	**MH635022**	-
	UFRN–Fungos 168 Paratype	Brazil	**MH635023**	-
	UFRN–Fungos 1803 Paratype	Brazil	**MH635024**	**MH635043**
**Outgroup—***G*. *velutinum*	MA-Fungi 83785	Argentina	KF988446	KF988581

The maximum parsimony (MP), maximum likelihood (ML), and Bayesian inference analyses are also deposited in protocols.io under the doi indicated above.

## Results

The ITS dataset included 48 sequences of *Geastrum* specimens, of which 34 were generated in this study (Table 1) and 14 obtained from EMBL/GenBank/DDBJ databases. In addition to the new species, sequences were obtained from type collections of five previously described species (Table 1).

The ITS alignment resulted in 657 unambiguously aligned nucleotide positions (317 constant, 103 parsimony-uninformative, and 237 parsimony-informative). MP analysis resulted in one most parsimonious tree (S1 Fig) with 862 steps (CI = 0.572, RI = 0.823, RC = 0.471, HI = 0.428). The ITS/LSU concatenated dataset included 28 samples with both ITS and LSU sequences, 1583 unambiguously aligned nucleotide positions: 1242 constant, 72 parsimony uninformative, and 269 parsimony-informative. Parsimony analyses resulted in a most parsimonious tree (S3 Fig) with 748 steps (CI = 0.591, RI = 0.779, RC = 0.460, HI = 0.409).

Maximum likelihood analyses with the default model GTRCAT gave a best ITS tree with—lnL = -2,900.087 (S2 Fig). Concatenated ITS/LSU likelihood analyses gave a best tree with—lnL = -4340.387 (S4 Fig).

The evolutionary models chosen by jModel-Test for Bayesian inference were HKY+G for ITS dataset (according to all criteria: AIC, BIC, AICc and DT), and TIM3+I+G for LSU dataset (according to AIC and AICc criteria—BIC and DT suggested TIM3+I, so we decided to use the more thorough algorithm). In the Bayesian analyses, the first 2,000 trees from the non-stationary phase were discarded. Maximum Clade Credibility tree and Posterior Probabilities (PP) were calculated from the 18,002 remaining trees. The summarized MCC tree has lnL = -2962.852. Concatenated ITS/LSU analysis gave us a MCC tree with lnL = -4329.812 (S5 Fig), which has been summarized from 18,002 trees after excluding 2,000 initial samples from the non-stationary phase.

**Fig 1:**
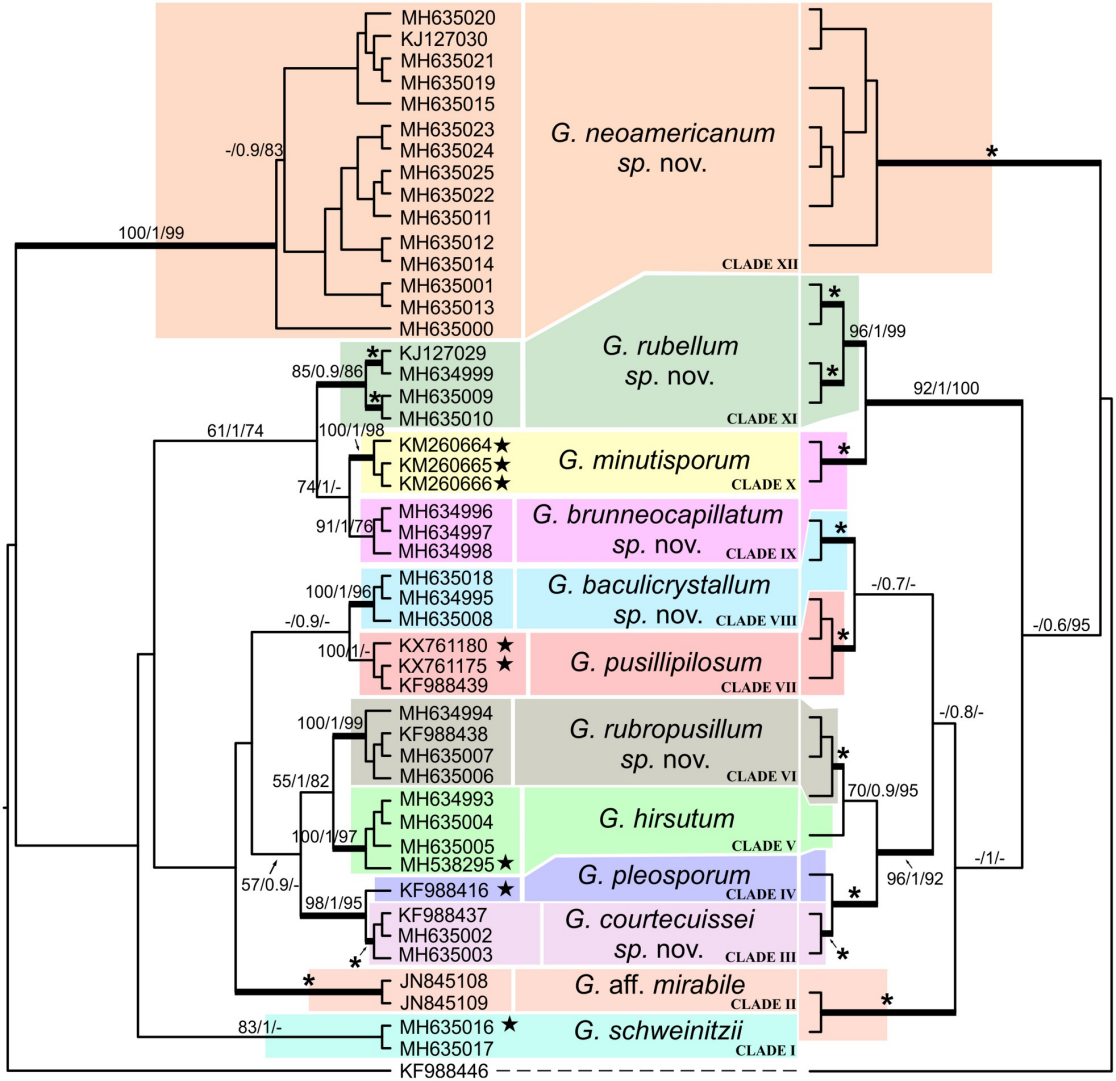
Bayesian trees of ITS (on the left) and ITS/LSU (on the right) nrDNA sequences of *Geastrum* species. *Geastrum velutinum* was used as out-group. Terminal branches are labeled with GenBank accession numbers. For further details, see Table 1. Numbers at the nodes indicate the Maximum Parsimony bootstrap, bootstrap values obtained from Maximum likelihood, and Posterior Probabilities from Bayesian analysis (MPbs /MLbs/ PP). Thick-lined branches are those with higher support (MPbs and MLbs higher than 85%, and PP higher than 0.85). Asterisk denotes fully supported branches in all three analyses.

Tree topologies generated by maximum parsimony (S1 and S3 Figs), maximum likelihood (S2 and S4 Figs), and Bayesian analyses (Fig 1 and S5 Fig) were similar, showing equivalent clustering patterns at terminal nodes. Except in ML analyses, *G*. *schweinitzii* isotype K(M) 180187 (seq. MH635016) and the sample INPA143435 (seq. MH635017) do not cluster together, and appear as singletons.

Excluding the outgroup, eleven delimited clades and a singleton (clade IV) are shown in Fig 1, corresponding to 12 species. The singleton is the isotype of *G*. *pleosporum* Douanla-Meli from Cameroon (Africa); among these delimited clades, six of them constitute new species:

Clade I. *Geastrum schweinitzii* isotype from Suriname (seq. MH635016), and a collection from Roraima-Brazil (seq. MH635017) grouped together. Specimens were collected in “Guiana Shield” biogeomorphological and pristine area of the Amazon forest, where there is a high level of endemism [40,41,42,43,44,45,46].

Clade II. The two sequences identified as *Geastrum* aff. *mirabile* Mont. obtained from GenBank (seqs. JN845108/JN845226 and JN845109/JN845227); [16]) formed a strongly supported group (MPbs = 100, MLbs = 100, PP = 1). According to the authors, specimens were collected in Asia (Bonin Island-Japan). In the protologue, these specimens of *G*. *mirabile*, are described as having small basidiomata, sessile endoperidial body, basidiospores 3.5–5.0 μm diam., and lignicolous habit. Leprieur’s revision of the original type collections of *G*. *mirabile* from French Guiana held at PC (PC0084351, Leprieur 849, 2 plates), not successfully sequenced, suggests that *G*. *mirabile* is a morphological synonym of *G*. *schweinitzii*. Since we have not been able to analyze the Japanese collections of Kasuya et al. [16] we cannot ensure whether their morphology matches that of the PC vouchers of *G*. *mirabile*. However, all available photographs of the samples alleged to be *G*. *mirabile* at on-line TNS fungarium databases (including source materials of the sequences used in our analysis) are from specimens with small mycelial tufts in a slightly hirsute exoperidium (S6 Fig). Thus, Japanese material from TNS claimed to be *G*. *mirabile* needs reassessment because they did not group in any of the clades presenting ‘*schweinitzii*-like’ morphology, and do not exhibit this morphological pattern. On the other hand, original PC vouchers can be either a synonym of *G*. *schweinitzii* or a cryptic species, and only molecular assessment of this material can clarify its real identity.

Clade III. The specimens LIP PAM/GUAD 11.04 (seq. MH635002) and LIP FH2004090503 (seqs. MH635003/MH635033) from the Caribbean (Guadeloupe Island, France), and MA-Fungi 83799 (under *Geastrum schweinitzii*; seqs. KF988437/KF988567) from South America (Argentina), grouped with maximum support (MPbs = 100, MLbs = 100, PP = 1). Both morphological, unique barcode sequence and concatenated ITS/LSU phylogenetic analysis lead us to describe a new species, *G*. *courtecuissei* sp. nov.

Clade IV (singleton). *Geastrum pleosporum* isotype (MA–Fungi 56971; seqs. KF988416/KF988544), from Africa (Cameroon), is the sister species of the previous one.

**Fig 2:**
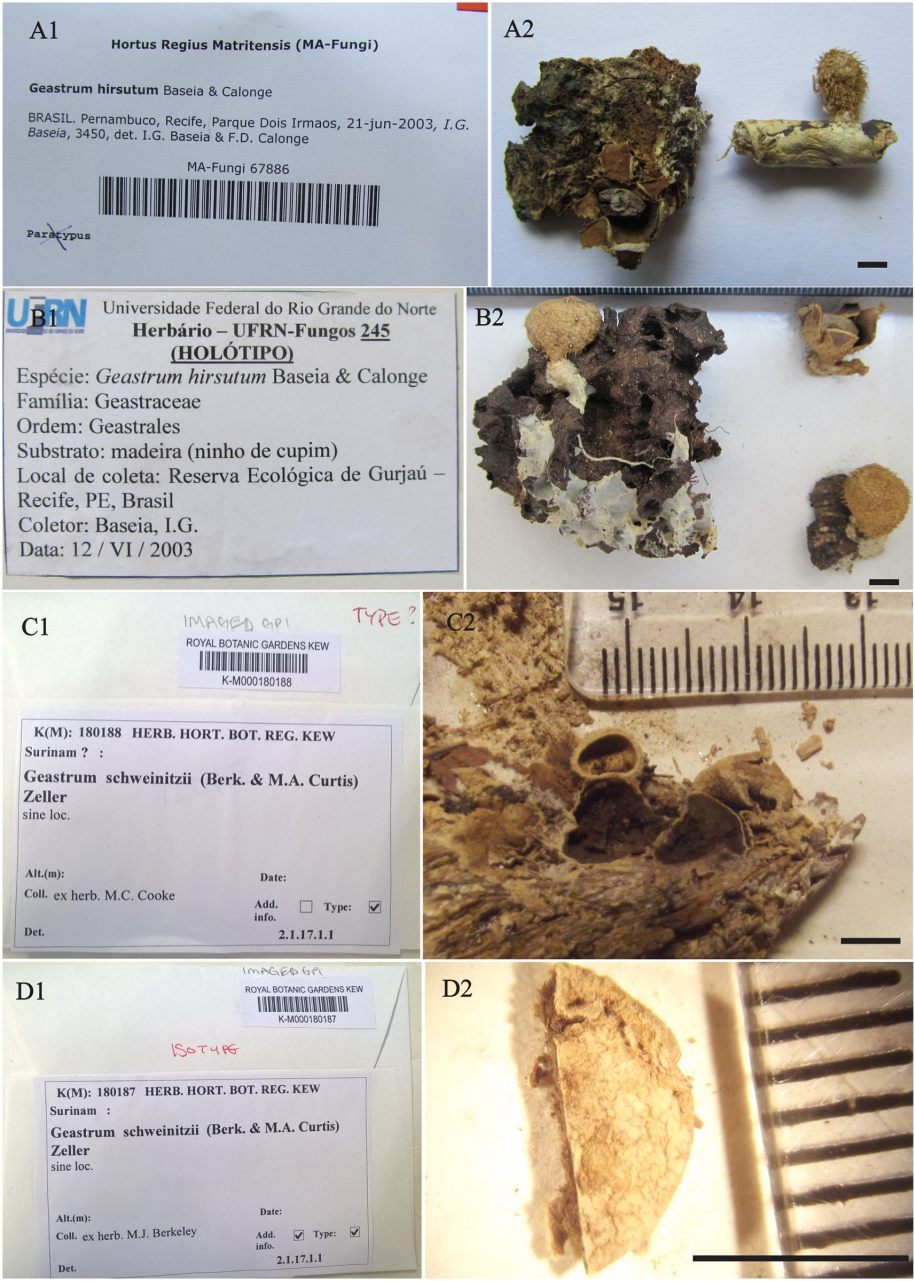
Type collections of *Geastrum hirsutum* and *Geastrum schweinitzii*. (A) *Geastrum hirsutum* MA-Fungi 67886, paratype. (B) *Geastrum hirsutum* UFRN-Fungos 245, holotype. (C) *Geastrum schweinitzii* K (M) 180188, type. (D) *Geastrum schweinitzii* K (M) 180187, isotype. Photo credits C, D: Donis Alfredo. A2, B2, C2, D2 bar = 5mm.

Clade V. Two collections from Brazil, including the paratype of *G*. *hirsutum* (MA-Fungi 67886; seq. MH538295, Fig 2) from Brazil, grouped with two collections, LIP MR/GUY 12.171 (seq. MH635004) and LIP RC/GUY 12.086 (seq. MH635005), from French Guiana. This clade is highly supported (MPbs = 100, MLbs = 97, PP = 1).

Clade VI. Four specimens, LIP RC/MART 03.015 (seq. MH635006), LIP CL/MART 08.112 (seqs. MH635007/MH635034), MA-Fungi 36141 (under *G*. *schweinitzii*, seqs. KF988438/KF988568) and UFRN 2308 (seqs. MH634994/MH635027), from Central America (Panama), the Caribbean (Martinique Island) and South America (Brazil), respectively, grouped together with strong support (MPbs = 100, MLbs = 99, PP = 1). Both morphological and unique barcode sequence led us to describe a new species, *G*. *rubropusillum* sp. nov.

Clade VII. The holotype UFRN-Fungos 2315 (seqs. KX761175/KX761176) and paratype UFRN-Fungos 2256 (seq. KX761180) of *G*. *pusillipilosum*, a recently published species [12], grouped together (MPbs = 100, MLbs = 30, PP = 1). They were collected in South America (Brazil and Argentina).

Clade VIII. A specimen from French Guiana, LIP RC/GUY 08.035 (seq. MH635008), and two from Brazil, UFRN-Fungos 1857 and 2835 (seqs. MH635018/MH635035 and MH634995/MH635028) joined together in a highly supported group (MPbs = 100, MLbs = 96, PP = 1). Both morphology and a unique barcode sequence led us to describe a new species, *G*. *baculicrystallinum* sp. nov.

Clade IX. Sample UFRN-Fungos 2851 (seq. MH634998) from Central America (Costa Rica), and samples UFRN-Fungos 2286 and 2834 (seqs. MH634996/MH635029 and MH634997/MH635030) from South America (Brazil) grouped together in a well-supported clade (MPbs = 91, MLbs = 76, PP = 1). Both morphology and a unique barcode sequence led us to describe a new species, *Geastrum brunneocapillatum* sp. nov.

Clade X. Three sequences of *G*. *minutisporum* from Argentina including the holotype (seqs. KM260664, KM260665 and KM260666) grouped together (MPbs = 100, MLbs = 98, PP = 1).

Clade XI. Two specimens, LIP CL/MART 08.067B (seqs. MH635009/MH635035) and LIP PAM/MART 12.100 (seq. MH635010/MH635037), from the Caribbean (Martinique Island), and two from South America (Brazil), UFRN-Fungos 1214 and 2844 (seqs. KJ127029/JQ683662 and MH634999/MH635031), came together in a well-supported group (MPbs = 85, MLbs = 86, PP = 0.9). Both morphology and unique barcode sequence led us to describe a new species, *G*. *rubellum* sp. nov.

Clade XII. Fifteen specimens from Brazil, French Guiana and Caribbean Islands clustered together in a highly supported group (MPbs = 100, MLbs = 99, PP = 1). Both morphology and unique barcode sequence led us to describe a new species, *G*. *neoamericanum* sp. nov.

The morphological ambiguity in type collections of *G*. *schweinitzii* K(M) 180187 (Fig 2) along with its not-fully-supported node among our molecular analyses lead us to create a new alignment with its clade (K(M) 180187 plus INPA 143435; seqs. MH635016 plus MH635017), and those species showing morphotypes in accordance with descriptions of *G*. *schweinitzii* (*G*. *neoamericanum* sp. nov., *G*. cf. *mirabile*, and *G*. *baculicrystallum*). The alignment resulted in 22 sequences (Table 1), with 441 nucleotide positions, and 326 constant nucleotides.

**Fig 3:**
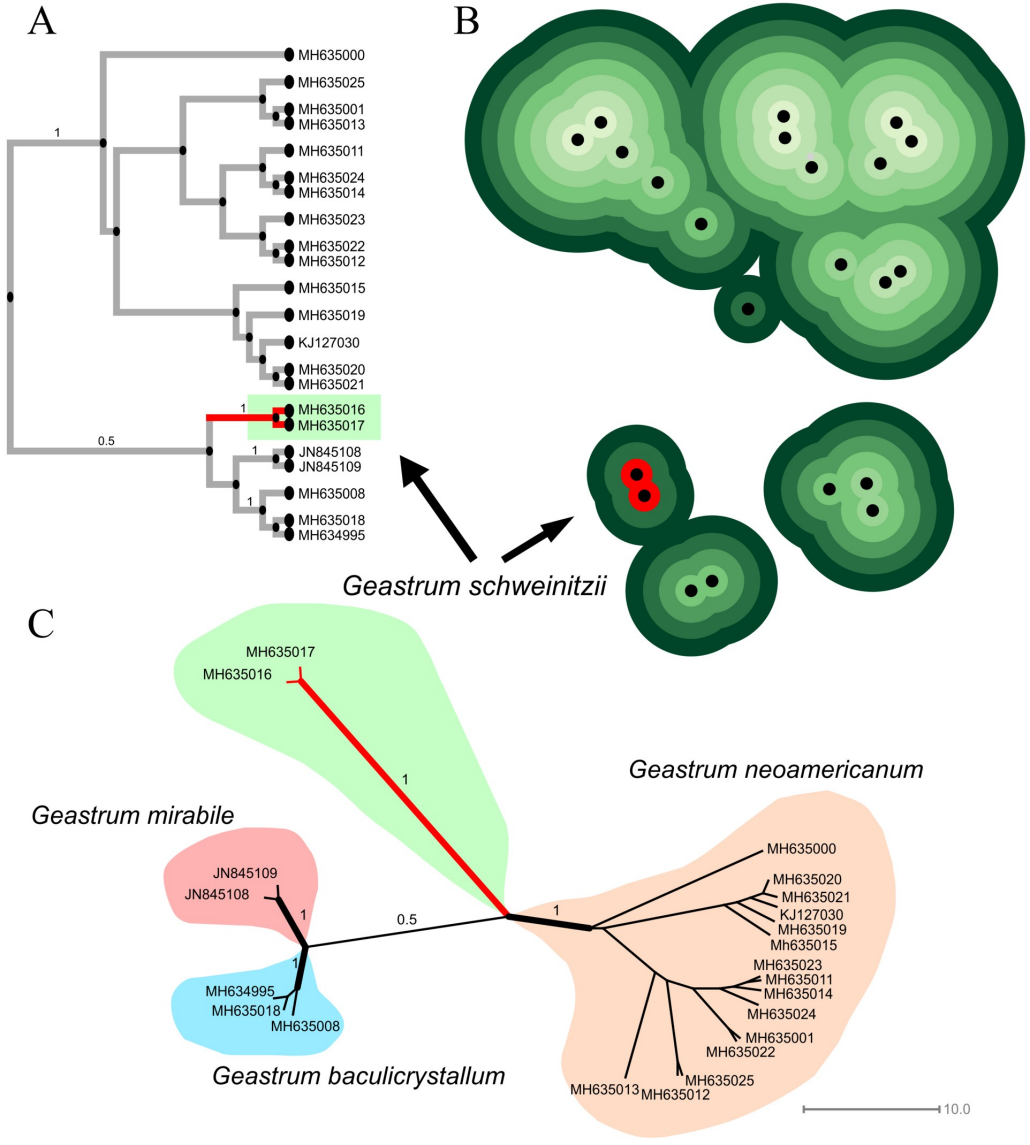
Analysis involving species of *Geastrum schweinitzii* complex. (A) Bayesian analysis conducted in Beast 2 software along 50 million generations. (B) Topo-phylogenetic and (C) phylogenetic network representations.

Bayesian analysis of this short alignment, conducted in Beast 2 software, along 50 million generations is shown in Fig 3a, along with Topo-Phylogenetic (Fig 3b) and Network representations (Fig 3c). The Maximum Clade Credibility tree summarized after discarding the non-stationary burn-in has lnL = -1291.281;also, Topo-Phylogeny and Phylogenetic Network clearly delimited the clade formed by INPA 143435 (seq. MH635017) plus K(M) 180187 (*G*. *schweinitzii* isotype; seq. MH635016) as a solid taxon.

### Taxonomy

#### *Geastrum baculicrystallum* J.O. Sousa, Accioly, Baseia & M.P. Martín, sp. nov., Fig 4, B1–B5 Mycobank MB 827089

**Fig 4:**
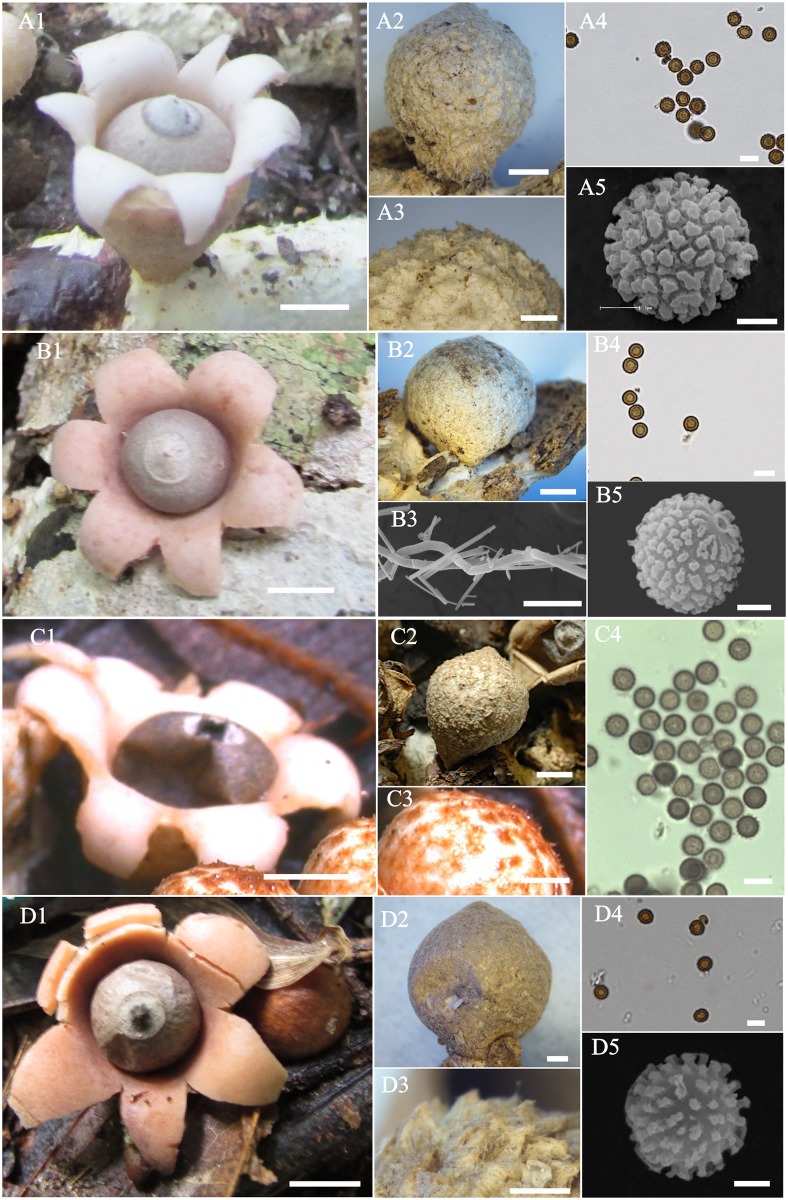
*Geastrum schweinitzii* and allies. (A). *Geastrum neoamericanum* sp. nov. (B) *Geastrum baculicrystallum* sp. nov. (C). *Geastrum courtecuissei* sp. nov. (D) *Geastrum rubropusillum* sp. nov. A1, B1, C1, D1 Expanded basidiomata. A2, B2, C2, D2 Unexpanded Basidiomata. A3, C3, D3 Exoperidium detail. B3. Rhizomorphs under SEM. A4, B4, C4, D4 Basidiospores under LM. A5, B5, D5 basidiospores under SEM. A1, B1, D1 bar = 5 mm; C1 bar = 3 mm; A2, B2, C2 bar = 2 mm; A3, D2 bar = 1 mm; A4, B3, C4, D4 bar = 5 μm; A5, B5, D5 bar = 1 μm.

Etymology. Referring to the rod-shaped crystals of the rhizomorphs.

Holotype. Brazil, Paraíba, Areia, Mata do Pau Ferro, 22 Jul. 2014, leg. J.O. Sousa, D.S. Alfredo & E.J. Souza, JM137 (UFRN–Fungos 2835, ITS sequence GenBank MH634995, LSU sequence GenBank MH635028).

Diagnosis. *Geastrum baculicrystallum* is very close to *G*. *neoamericanum* sp. nov. in morphology; however, *G*. *baculicrystallum* is differentiated by the cylindrical (2.3×0.9 mm), smaller warts of basidiospores (0.02–0.08 μm high), inconspicuous in light microscope, and presence of cystidia in subiculum hyphae.

Description. Unexpanded basidiomata epigeous, brownish grey (5C2 to 5B2), citriform, 6–8 × 5–8 mm, surface tomentose to rugose when mature, not encrusted with debris. Subiculum white (4A1), covering the substrate, with exudate. Expanded basidiomata saccate, 5–7 mm × 8–10 mm wide. Exoperidium splitting into 5–6 revolute, 3–4 mm diam, triangular rays, non–hygroscopic. Mycelial layer yellowish white (4A2), surface papery, rugose, not encrusted with debris, persistent. Fibrous layer white orange (5A2), papery. Pseudoparenchymatous layer light brown (5D4), persistent. Endoperidial body grayish brown (6E3) subglobose to depressed globose, 2–4 × 3–6 mm, sessile, glabrous. Peristome fimbriate, delimited by a line, grayish brown (6D3), lighter than endoperidium, mammiform, depressed on endoperidium. Columella cylindrical, 2.3 × 0.9 mm, central, inconspicuous, white (4A1).

Subiculum composed of hyaline, filamentous, slender, hyphae, 0.6–1.0 μm diam, dextrinoid, sinuous, crystals not seen, presence of cystidia. Rhizomorphs composed of hyaline hyphae, surface covered by crystals with narrow oblique prism shape, 24.2–41.7 × 8.4–12.3 μm. Mycelial layer formed of hyaline hyphae, thin-walled (0.5–0.9 μm diam), 3.1–7.0 μm diam, dextrinoid, surface encrusted, lumen slightly evident, branched. Fibrous layer formed of hyaline hyphae, 5.4–9.2 μm diam, surface non–encrusted, lumen evident. Pseudoparenchymatous layer formed by brownish hyphal cells, subglobose to oval, 30.4–34.9 × 21.1–30.1 μm, thin–walled (0.6–0.9 μm). Eucapillitium brownish, thick-walled (0.4–0.8 μm diam), 2.6–4.7 μm diam, surface slightly encrusted, lumen not evident. Basidia, yellowish, thin to thick walls (0.4–1.2 μm), subglobose to oval, 9.4–16.4 × 6.4–10.0 μm. Basidiospores brownish, subglobose, 3.8–4.5 × 3.6–4.4 μm (x = 4.1 ± 0.2 × 3.9 ± 0.2, Q_m_ = 1.04, n = 30), ornamentation inconspicuous under LM, verrucose under SEM, warts very small (0.02–0.08 μm high).

Ecology and distribution. This species has a Neotropical distribution. Found in the biome Tropical & Subtropical Moist Broadleaf Forests of Brazil (Pernambuco coastal forests ecoregion), and French Guiana (Guianan lowland moist forests) on decaying wood with gregarious or caespitose growth.

Additional material examined. Brazil, Paraíba, Areia, Mata do Pau Ferro, 17 Jul. 2012, leg. D.S. Alfredo (UFRN–Fungos 1857 paratype, ITS sequence GenBank MH635018, LSU sequence Genbank MH635035). French Guiana, Saül, Layon des Grands Arbres, 3 May 2008, leg. R. Courtecuisse (LIP RC/GUY 08.035, ITS sequence GenBank MH635008).

Remarks. This species is basically characterized by small basidiomata (8–10 mm wide when expanded), exoperidium tomentose to rugose; peristome delimited by a grayish brown line, depressed on the endoperidium; columella cylindrical (2.3 × 0.9 mm); basidiospores globose to subglobose 3.6–4.5 μm diam, presence of cystidia on subiculum hyphae. It is morphologically related to *G*. *neoamericanum*. However, there are small differences between them: the basidiospores of *G*. *neoamericanum* are slightly larger (3.8–5.1 × 3.7–4.9 μm), with longer warts; the basidia differ, with clavate, pyriform to lageniform shapes in *G*. *neoamericanum*, and the crystals with rod shape in *G*. *baculicrystallum*. The bacullar crystals on the rhizomorphs, and more inconspicuous warts on the basidiospores in *G*. *baculicrystallum* can differentiate this species from *G*. *schweinitzii*; we can consider *G*. *baculicrystallum* a semi-cryptic species of *G*. *schweinitzii*.

#### *Geastrum brunneocapillatum* J.O. Sousa, Accioly, M.P. Martín & Baseia, sp. nov., Fig 5, A1–A5, Mycobank MB 827086

**Fig 5:**
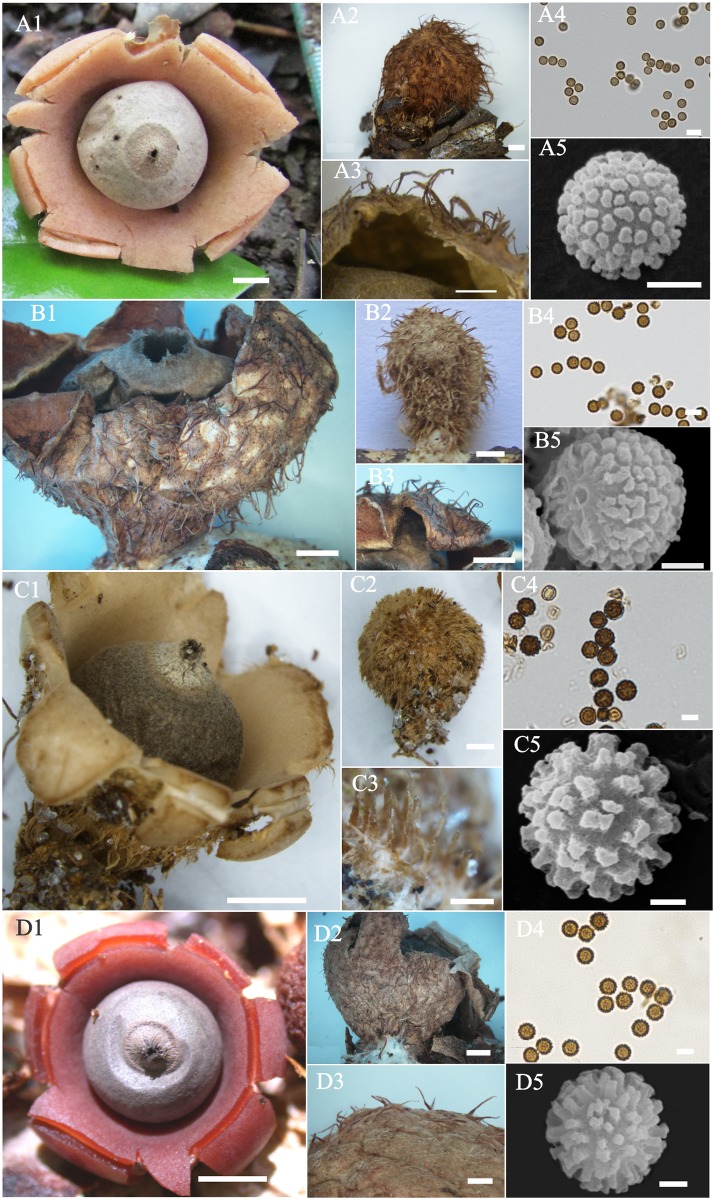
*Geastrum hirsutum* and allies. (A) *Geastrum brunneocapillatum* sp. nov. (B) *Geastrum hirsutum*. (C) *Geastrum pusillipilosum*. (D) *Geastrum rubellum* sp. nov. A1, B1, C1, D1 Expanded basidiomata. A2, B2, C2, D2 Unexpanded Basidiomata. A3, B3, C3, D3 Exoperidium detail. A4, B4, C4, D4 Basidiospores under LM. A5, B5, C5, D5 basidiospores under SEM. A1 bar = 2 mm; A2, A3, C2, bar = 1 mm; B1, B2, B3, C1, D2 bar = 2 mm; C3 bar = 0.1 mm; D1 bar = 4 mm; A4, B4, C4, D4 bar = 5 μm; A5, B5, C5, D5 bar = 1 μm.

Etymology. Referring to the dark brown hairs on the exoperidium.

Holotype. Paraíba, Mamanguape, Reserva Biológica de Guaribas, 26 Jun. 2014, leg. J.O. Sousa, Y.M. Nascimento & E.J. Souza JM101 (UFRN–Fungos 2286, ITS sequence GenBank MH634996, LSU sequence GenBank MH635029).

Diagnosis. This species is morphologically close to *G*. *pusillipilosum*; but the sparsely hairy mycelial layer, longer exoperidium hairs (0.5–2.0 mm high) and smaller basidiospores (2.7–4 μm diam) in *G*. *brunneocapillatum*, clearly differentiates these species. Also *G*. *rubellum* resembles *G*. *brunneocapillatum*, but the latter has longer (0.5–2.0 mm high) hairs and smaller basidiospores 2.8–4.0 μm diam.

Description. Unexpanded basidiomata epigeous, brown (5E5) to brownish orange (5C4), subglobose, pyriform, obpyriform to oval, 7–13 × 6–10 mm, surface hairy, not encrusted. Subiculum orange white (6A2) under some cespitose basidiomata. Expanded basidiomata saccate, 4.1–11 mm high × 8–26 mm wide. Exoperidium splitting into 5–7 revolute, triangular rays, non–hygroscopic. Mycelial layer yellowish (5D4), not encrusted with debris, persistent or peeling away in irregular patches. Hairs dark brown, long (0.5–2 mm high), ephemeral when fully mature. Fibrous layer yellowish white (4A2), papery. Pseudoparenchymatous layer yellowish (5D4), persistent, glabrous. Endoperidial body globose, 4–9 × 3–11 mm, sessile, glabrous, grey orange (5B2). Columella cylindrical, 4.9 × 1.7 mm, central, inconspicuous, grey orange (6B2). Peristome finely fibrillose, delimited, delimitation yellowish (5D4), darker than endoperidium. Gleba dark brown (6F4).

Subiculum composed of hyaline, filamentous, slender, hyphae, 0.9–1.0 μm diam, dextrinoid, sinuous. Subiculum and rhizomorphs with surface covered by crystals, with coarser and more irregular oblique prism shape. Mycelial layer composed of hyaline to yellowish, thin–walled hyphae (<1 μm), 1.66–3.04 μm diam, dextrinoid, lumen not evident. Hairs dark brown, thick–walled (0.7–1.0 μm.), skeletal hyphae, 3.2–4.5 μm diam, strongly dextrinoid, sinuous, lumen not conspicuous. Fibrous layer composed of hyaline, sinuous, thick–walled hyphae (0.7–1.15 μm), 3.8–7.6 μm diam, encrusted, branched, lumen evident. Pseudoparenchymatous layer composed of light brown, thin–walled (0.75–1.48 μm) hyphal cells, oval, ellipsoid, lageniform to pyriform, 30.5–65.6 × 21.1–55.5 μm. Eucapillitium yellowish brown, thin walls (0.5–1.0 μm), 1.5–3.8 μm diam, encrusted or not, lumen evident. Basidia, yellowish, thin-walled (0.4–0.7 μm), clavate, pyriform to lageniform, 12.2–19.3 × 3.8–6.7 μm. Basidiospores yellowish, globose to subglobose, 2.8–4.0 × 2.7–4.0 μm (x = 3.3 ± 0.4 × 3.2 ± 0.4, Q_m_ = 1.03, n = 90), ornamentation inconspicuous under LM, verrucose under SEM; warts short (up to 0.4 μm high), rounded tips, apiculus reduced.

Ecology and distribution. This species has a Neotropical distribution. Found in the biome Tropical & Subtropical Moist Broadleaf Forests of the Brazil (Pernambuco coastal forests and Pernambuco interior forests ecoregions) and Costa Rica (Isthmian-Atlantic moist forests ecoregion) on soil covered by leaf–litter, decaying wood or termites nest, with gregarious or caespitose growth.

Additional material examined. brazil, Paraíba, Areia, Mata do Pau Ferro, Trilha Boa Vista, 21 Jul. 2014, leg. J.O. Sousa, D.S. Alfredo & E.J. Souza, JM117 (UFRN–Fungos 2834, paratype, ITS sequence GenBank MH634997). costa rica, La Selva Biological Station, C. Ovrebo 2303 (UFRN–Fungos 2851, paratype, ITS sequence GenBank MH634998).

Additional species examined. *Geastrum hirsutum*, Brazil–Pernambuco, Recife, Reserva Ecológica do Gurjaú, 12 Jul. 2003, leg. I.G. Baseia (MA-Fungi 67886, paratype, ITS sequence GenBank MH538295) (Fig 5, B1–B5); Amazonas, Manaus, Reserva Florestal Adolfo Ducke, 02 Jul. 2014, leg. M.D.F. Santana (INPA 259950, ITS sequence GenBank MH634993 and LSU sequence GenBank MH635026). *Geastrum pusillipilosum*, Brazil. Paraíba, Mamanguape, REBIO Guaribas, SEMA II, 26 Jun. 2014, leg. J.O. Sousa JM100 (UFRN–Fungos 2315, holotype, ITS sequence GenBank KX761175 and LSU sequence GenBank KX761176) (Fig 5, C1–C5); Ceará, Crato, Floresta Nacional do Araripe, 01 Apr. 2014, leg. R.J. Ferreira (UFRN–Fungos 2256, paratype, ITS sequence GenBank KX761180); Paraíba, Mamanguape, Reserva Biológica Guaribas, 11 Jul. 2015, leg. J.O. Sousa, J.F. Freitas-Neto (UFRN–Fungos 2759, ITS sequence GenBank KX761177 and LSU sequence GenBank KX761178).

Remarks. This species is recognized by its yellowish exoperidium; with ephemeral, long (0.5–2.0 mm high), dark brown hairs; peristome delimited by a yellowish line; and basidiospores globose to subglobose, 2.8–4.0 μm diam. It is morphologically close to other species with hairy exoperidium, such as *G*. *pusillipilosum* and *G*. *rubellum*; however, these four species are fundamentally distinct by their larger basidiospores (5.0–6.5 μm diam and 4.4–6.3 μm diam., respectively). Additionally, *G*. *pusillipilosum* and *G*. *rubellum* have smaller hairs in the exoperidium (up to 0.3 mm; and up to 0.68 mm longer, respectively) than *G*. *brunneocapillatum*. We can consider *G*. *brunneocapillatum* a semi-cryptic species of *G*. *hirsutum* (Fig 5, B1–B5), since the latter species has slightly larger basidiospores 4.1–4.9 μm. Another species with morphological features similar to *G*. *brunneocapillatum* is *G*. *minutisporum* described without subiculum and with very tiny basidiospores (2–)2.5–3 mm diam [47].

#### *Geastrum courtecuissei* P.-A. Moreau, C. Lécuru, sp. nov., Fig 4, C1–C5 Mycobank MB 827090

Etymology. In honor to Dr. Courtecuisse, research scientist from France.

Holotype. Guadeloupe, Saint-Claude, Matouba, along the Victor Hugues trail, on litter of *Cupressus* cf. *macrocarpa*, leg. F. Hairie, 9 Sep. 2004 (LIP FH2004090503, ITS sequence GenBank MH635003, LSU sequence GenBank MH635033).

Diagnosis. This species is similar to *G*. *baculicrystallum*, but *G*. *courtecuissei* is distinguished by the presence of echinulate exoperidium and basidiospores with longer warts, conspicuous under LM. Another morphologically closel-related species is *G*. *pleosporum*, which has polymorphic basidiospores (4.0–7.0 × 3.2–5.0 μm), while, *G*. *baculicrystallum* has globose to subglobose basidiospores (3.7–4.9 μm diam).

Description. Unexpanded basidiomata epigeous, light brown (N_10_Y_30_M_10_), subglobose to citriform, 2.5–22 × 4.1–20 mm, surface slightly tomentose to rugulose, papery to cottonous, not encrusted. Subiculum whitish (N_10_Y_20_M_10_), covering the substrate. Expanded basidiomata saccate, 2.8–8.1 mm high × 4.8–35 mm wide. Exoperidium splitting into 4–8 revolute rays, non–hygroscopic. Mycelial layer at first echinulate with short, whitish hairs, easily broken, forming collapsed ochre–brown patches and veins on whitish ground, light brown (N_10_Y_40_M_10_), not encrusted. Fibrous layer papery, white (N_00_Y_10_M_00_). Pseudoparenchymatous layer orange brown (N_00_Y_40_M_10_), peeling-off in irregular patches. Endoperidial body surface steel grey when fresh, brown (N_30_Y_30_M_20_) when dry, subglobose, 1.5–2.6 × 3.0–15 mm sessile, covered with a persistent whitish tomentum. Peristome fimbriate, delimited by light brown line (N_20_Y_20_M_10_), lighter or darker than endoperidium, mammiform to papilla-like, < 1 mm high, 2–2.5 mm wide, depressed on endoperidium. Columella not seen. Mature gleba greyish brown (N_70_Y_20_M_10)_

Subiculum composed of hyaline, filamentous, slender hyphae, <1–1.8 μm diam, dextrinoid, strongly sinuous, thick–walled, sometimes coiled; in depth, made of more or less parallel slender hyphae 1–2.5 μm diam, with sparse broader skeletal hyphae 2.3 μm diam, all smooth and colorless, slightly dextrinoid; with crystals coarser and more irregular oblique prisms. Rhizomorphs composed of hyaline, slender hyphae, 1.0–3.0 μm diam, lumen not evident, some with clamps and ampulliform septa, surface covered by crystals, with narrow oblique prism shape. Mycelial layer formed of hyaline, thick-walled (0.5–3.5 μm diam) hyphae, 2.9–6.0 μm diam, dextrinoid, surface not encrusted, lumen evident, not branched, some with yellow granular content; terminations rounded to attenuate, smooth, always with yellow content. Fibrous layer formed of hyaline, thin-walled (1.0–1.1 μm diam) hyphae, 3.9–7.5 μm diam, surface encrusted, lumen evident. Pseudoparenchymatous layer formed by hyaline to yellowish hyphal cells, subglobose, ellipsoid to pyriform, 19.5–37 × 18.0–36.5 μm, thin–walled (0.8–1.0 μm diam). Eucapillitium light brown, 2.5–3.9 μm diam, thin–walled (<1 μm diam), surface not encrusted, no lumen evident. Basidia cylindrical, pyriform to elongated 9–20 × 2–6.5 μm, 2–4 sterigmata. Basidiospores brownish, globose to subglobose, 3.8–5.0 × 3.7–4.9 μm (x = 4.1 ± 0.4 × 4.0 ± 0.3, Q_m_ = 1.03, n = 30), ornamentation conspicuous in LM.

Ecology and distribution. This species has a Neotropical distribution. Found in the biome Tropical & Subtropical Moist Broadleaf Forests of Argentina (Alto Paraná Atlantic forest ecoregion) and Guadeloupe Island (Leeward Islands moist forest ecoregion).

Additional material examined. Argentina, Puerto Iguazú, *no date*, leg. E. Grassi (MA–Fungi 83779, paratype, ITS sequence GenBank KF988437, LSU sequence GenBank KF988567). Guadeloupe, Saint–Claude, Matouba, along the Victor Hugues trail, on litter of *Cupressus* cf. *macrocarpa*, 10 Aug. 2011, leg. P.-A. Moreau, LIP PAM/GUAD 11.04, paratype, ITS sequence GenBank MH635002).

Remarks. This species has an exoperidium layer at first echinulate, forming collapsed ochre–brown patches and veins on whitish ground when fully mature; peristome delimited by a light brown line, depressed on endoperidium; basidiospores globose to subglobose with 3.8–5.0 μm diam. It resembles *G*. *pusillipilosum*, however, *G*. *pusillipilosum* has a densely hairy exoperidium and larger basidiospores (5.0–6.5 μm diam). *Geastrum schweinitzii*, and *G*. *neoamericanum* are other species with very closely related morphology, but these species do not have the echinulate exoperidium. *Geastrum pleosporum* is very close to *G*. *courtecuissei* in the phylogenetic tree, but *G*. *pleosporum* differs in having a reddish pseudoparenchymatous layer, polymorphic basidiospores (4.0–7.0 × 3.2–5.0 μm) with smooth to nearly smooth ornamentation.

#### *Geastrum neoamericanum* J.O. Sousa, Accioly, M.P. Martín & Baseia, sp. nov., Fig 4, A1–A5, Mycobank MB 827091

Etymology. Referring to the widespread occurrence in Latin America.

Holotype. Brazil, Paraíba, Mamanguape, REBIO Guaribas, SEMA II, 25 Jun. 2014, J.O. Sousa, Y.M. Nascimento & E. Souza JM90 (UFRN–Fungos 2302, ITS sequence GenBank MH635001, LSU sequence GenBank MH635040).

Diagnosis. This species is morphologically similar to *Geastrum baculicrystallum*, but *G*. *neoamericanum* is differentiated by ampuliform columella 4.5×0.6 mm, longer warts of basidiospores (0.43–0.91 μm high), conspicuous in light microscope, and absence of cystidia in subiculum hyphae.

Description. Unexpanded basidiomata epigeous, yellowish white (4A2), citriform to lacrimiform, 5–9 × 3–8 mm, surface tomentose to rugulose when mature, not encrusted. Subiculum white (4A1), covering the substrate, producing exudate in some basidiomata. Expanded basidiomata saccate, 3–8 mm high × 8–10 mm wide. Exoperidium splitting into 5–6 revolute, triangular rays, 2–3 mm diam, non–hygroscopic. Mycelial layer yellowish white (4A2) to orange white (5A2), surface rugose, tomentose to papery when mature, not encrusted, persistent. Fibrous layer papery, white (4A1). Pseudoparenchymatous layer brown (6D4) to orange grey (5B4), persistent. Endoperidial body greyish brown (6F3) to orange grey (5C3), subglobose to depressed–globose, 3–5 × 4–6 mm sessile, glabrous. Peristome fimbriate, with folds on apex, delimited by a line brownish grey (6F2) to greyish brown (5C3), lighter than endoperidium, mammiform, < 1 mm high. Columella ampuliform, 4.5 × 0.6 mm, central, inconspicuous, orange white (5A2).

Subiculum composed of hyaline, filamentous, slender hyphae, 0.6–1.7 μm diam, dextrinoid, strongly sinuous. Rhizomorphs composed of hyaline, slender hyphae, 1.2–2.5 μm diam, surface encrusted, lumen not evident, surface covered by crystals, with narrow oblique prism shape. Mycelial layer formed of hyaline, thick-walled (0.5–1.2 μm diam) hyphae, 5.3–10.1 μm diam, dextrinoid, surface not encrusted, lumen evident, branched apex. Fibrous layer formed of hyaline, thick-walled (0.6–1.3 μm diam) hyphae, 5.1–9.5 μm diam, surface encrusted, lumen non–evident. Pseudoparenchymatous layer formed by brownish hyphal cells, subglobose to pyriform, 32.6–59.5 × 23.4–40.8 μm, thick–walled (0.9–1.8 μm diam). Eucapillitium dark brown, 2.7–4.0 μm diam, thin–walled (0.5–0.8 μm diam), surface slightly encrusted, no lumen evident. Basidia yellowish, thin-walled (0.4–0.7 μm), clavate, pyriform to lageniform, 9.2–18.7 × 7.5–15.5 μm. Basidiospores brownish, globose to subglobose, 3.8–5.1 × 3.7–4.9 μm (x = 4.3 ± 0.3 × 4.2 ± 0.3, Q_m_ = 1.03, n = 90), ornamentation conspicuous in LM, densely verrucose under SEM, medium warts (0.43–0.91 μm high) with planar tips.

Ecology and distribution. This species has a Neotropical distribution. Found in the biome Tropical & Subtropical Moist Broadleaf Forests of the Brazil (Alto Paraná Atlantic forests, Bahia coastal forests, Pernambuco coastal forests, Pernambuco interior forests, Uatumã-Trombetas moist forests) and the Guiana shield (Suriname and French Guiana, Guyanan lowland moist forest ecoregion), and in the Caribbean (Guadeloupe, Martinique). Cespitose basidiomata grow on abundant subiculum, on decaying wood.

Additional specimens examined. Brazil, Paraíba, Areia, Mata do Pau Ferro, Trilha Engenho Triunfo, 18 Jul. 2013, J.O. Sousa & D.S Alfredo, JM64 (UFRN–Fungos 2149, paratype, ITS sequence GenBank MH635021, LSU sequence GenBank MH635039); Rio Grande do Norte, Parnamirim, Mata da Base Aérea de Natal, 23 Aug. 2007, leg. E.P. Fazolino (UFRN–Fungos 348, paratype, ITS sequence GenBank MH635019); Rondônia, Módulo Abunã, 25 Jun. 2014, leg. M.D.F. Santana (INPA 259949, paratype, ITS sequence GenBank MH635025, LSU sequence GenBank MH635041); Minas Gerais, Santa Rita do Sapucaí, Reserva Biológica Municipal Santa Rita Mítzi Brandão, 2015, leg. D.S. Alfredo & P. Lavor, DSA279 (UFRN–Fungos 2850, paratype, ITS sequence GenBank MH635000, LSU sequence GenBank MH635032); Bahia, Ilhéus, RPPN Mãe da Mata, 19 Dec. 2006, leg. J.L. Bezerra 858 (CEPEC 1391, paratype, ITS sequence GenBank MH635020). Guadeloupe, Petit-Bourg, Carrère, Forêt départementalo-domaniale de Bois Sergeant, 21 Nov. 2006, leg. C. Lécuru (LIP CL/Guad 06.010, paratype, ITS sequence GenBank MH635011); Petit-Bourg, Route forestière de Jules, domaine de Duclos, 24 Nov. 2006, leg. C. Lécuru (LIP CL/Guad 06.056, paratype, ITS sequence GenBank MH635012). Martinique, Saint-Esprit, morne David, 23 Aug. 2004, leg. C. Lécuru (LIP CL/Mart 04.001, paratype, ITS sequence GenBank MH635013). French Guiana, 1 Mar. 2012, leg. J.-L. Cheype (LIP JLC12030103, paratype, ITS sequence GenBank MH635014, LSU sequence GenBank MH635038); 2012, leg. M. Roy (LIP MR–GUY–12–004, paratype, ITS sequence GenBank MH635015).

Remarks. This species is mainly characterized by small basidiomata (9–10 mm wide when expanded); exoperidium whitish to yellowish and rugose to tomentose; pseudoparenchymatous layer brown to orange grey; peristome mammiform, delimited by a brownish grey to greyish brown line; columella ampuliform; basidiospores globose to subglobose (Q_m_ = 1.03), 3.7–5.1 μm diam, with medium warts (0.43–0.91 μm high). It is a semi-cryptic species with *G*. *schweinitzii*, being distinct only by its slightly larger basidiospores 3.7–5.1 μm diam (against 3.5–4.2 μm in *G*. *schweinitzii*), and by longer warts on basidiospores (0.43–0.91 μm high), which are conspicuous under LM (against inconspicuous in *G*. *schweinitzii*). It also resembles *G*. *rubropusillum*, but the darker mycelial and pseudoparenchymatous layers (brownish and reddish, respectively), peristome depressed on the endoperidium, and subglobose to oval basidiospores (Q_m_ = 1.07) distinguish *G*. *rubropusillum* from *G*. *neoamericanum*.

#### *Geastrum rubellum* P.-A. Moreau & C. Lécuru. sp. nov., Fig 5, D1–D5, Mycobank MB 827092

Etymology. Named in reference to its reddish-coloured exoperidum.

Holotype. Martinique, Le Prêcheur, Anse Couleuvre, path towards Anse Lévrier, 17 Aug. 2012, leg. P.–A. Moreau (LIP PAM/Mart 12.100, ITS sequence GenBank MH635010, LSU sequence GenBank MH635037).

Diagnosis. This species is morphologically similar to *G*. *pusillipilosum* (Fig 2, C1–C5), but the smooth and sparsely hairy mycelial layer, longer exoperidium hairs (0.34–0.68 mm high), grayish endoperidium, and reddish pseudoparenchymatous layer in *G*. *rubellum*, clearly differentiate these species. Also *G*. *rubellum* resembles *G*. *brunneocapillatum*, but *G*. *rubellum* has reddish to brownish exoperidium with smaller hairs.

Description. Unexpanded basidiomata, globose to slightly attenuate at base, somewhat umbonate (more conspicuously when sectioned) before opening, 5–12 mm wide. Expanded basidiomata saccate, 10 mm high × 8.5–30 mm wide. Exoperidium splitting into 5–7 rays, involute or revolute, sometimes bifid, non–hygroscopic. Subiculum white (4A1) to yellowish white, found only on basidiomata base or widely spread. Mycelial layer pale pinkish white at first then becoming pinkish red, brown (5E6) when dried, surface not encrusted, rugose, slightly hirsute and persistent. Hairs concolorous with mycelial layer, spaced apart, 0.34–0.68 mm high, becoming vinaceous pink–red when collapsing. Fibrous layer pinkish gray when fresh, then yellowish white (4A2), papery. Pseudoparenchymatous layer purplish pink when fresh, brownish grey (6D3) when dried, persistent. Endoperidial body grey (6D3), subglobose, 6 × 3–10 mm, sessile, surface glabrous. Peristome fimbriate, delimited by a line, orange grey (6B2), mammiform, concolorous with endoperidium. Columella distinct, intruding 2/3 into the glebal mass, white, cylindrical. Mature gleba dark brown (6F4).

Subiculum composed of hyaline, filamentous, slender hyphae, dextrinoid, 0.9–1.6 μm diam, sinuous, presence of abundant crystals, with coarser and more irregular oblique prism shape, often clustered in rosette–like aggregates, mostly on surfaces but also internal, 3–25 μm long. Mycelial layer composed of hyaline, thin–walled hyphae (0.5–0.7 μm), 2.3–4.5 μm diam, dextrinoid, sinuous, surface not encrusted, lumen inconspicuous. Hairs composed of thick–walled (0.5–1.2 μm), brownish, skeletal hyphae, 4.7–6.4 μm diam, strongly dextrinoid, surface not encrusted, lumen not evident or slightly evident. Fibrous layer composed of hyaline, sinuous, thick–walled hyphae (0.6–0.9 μm), 4.3–7.2 μm diam, slightly encrusted, lumen evident. Pseudoparenchymatous layer composed of light brown, thin–walled (0.8–1.3 μm) hyphal cells, subglobose to oval, 33.7–57.0 × 41.4–37.1 μm. Eucapillitium brownish, thick walls (0.5–1.1 μm), 2.3–5.5 μm diam, surface encrusted, lumen evident. Basidia globose before maturity, then vesiculose to lageniform with a subcapitate neck bearing the insconspicuous sterigmata, 6.5–12 x 5–6.8 μm. Basidiospores dark brown, globose to subglobose, (3.5) 4.4–6.3 × 4.4–6.0 μm (x = 5.5 ± 0.5 × 5.3 ± 0.1, Q_m_ = 1.03, n = 60), ornamentation conspicuous under LM, warts 0.6–1.2 μm high.

Ecology and distribution. This species has a Neotropical distribution. Found in the biome Tropical & Subtropical Moist Broadleaf Forests of Brazil (Atlantic Coast restingas and Southwest Amazon moist forests ecoregions), and Martinique Island (Windward Islands moist forests ecoregion), growing in groups on soil covered by leaf–litter or decaying wood.

Additional material examined. Brazil. Rio Grande do Norte, Baía Formosa, Reserva Particular do Patrimônio Natural Mata Estrela, 09 Jun. 2009, leg. B.D.B Silva, I.G. Baseia, T.S. Cabral (UFRN–Fungos 1214, paratype, ITS sequence GenBank KJ127029, LSU sequence GenBank JQ683662); Acre, Floresta Nacional do Macauã, 29 Jan. 2016, leg. A.M. Ottoni, AMO 604 (UFRN–Fungos 2844, paratype, ITS sequence GenBank MH634999, LSU sequence GenBank MH635031). Martinique, Le Prêcheur, Anse Couleuvre, along the road, in secondary mesophytic forest, 25 Aug. 2008, leg. C. Lécuru (LIP CL/MART 08.067B, paratype, ITS sequence GenBank MH635009, LSU sequence GenBank MH635035).

Remarks. This species is recognized by the reddish to brown exoperidium in fully mature basidiomata, and verrucose to hairy mycelial layer, with ephemeral, short (0.34–0.68 mm high) sparse hairs; grey endoperidium; peristome delimited by an orange grey line; and basidiospores globose to subglobose, 4.4–6.3 μm diam. The morphology of *G*. *rubellum* is very similar to *G*. *hirsutum* and taxonomic misunderstandings can occur, as happened with the collection UFRN–Fungos 1214 (seq. KJ127029/JQ683662), which was previously determined as *G*. *hirsutum* [44]. However, *G*. *hirsutum* is distinguished by lighter endoperidium (grey orange 5B2), and by longer (0.6–1.8 mm high) and darker (dark brown) hairs on the exoperidium. The collection UFRN–Fungos 1214 is described here as *G*. *rubellum* sp. nov.

#### *Geastrum rubropusillum* J.O. Sousa, Accioly, M.P. Martín & Baseia, sp. nov., Fig 4, D1–D5, Mycobank MB 827094

Etymology. Referring to the reddish pseudoparenchymatous layer and the small size of basidiomata.

Holotype. Martinique, Le Prêcheur, anse Couleuvre, 1 Sep. 2003, leg. R. Courtecuisse (LIP RC/MART 03.015, ITS sequence GenBank MH635006).

Diagnosis. This species is morphologically close to *Geastrum pleosporum*, but *G*. *rubropusillum* is distinguished by its mammiform peristome and subglobose to oval basidiospores, (3.8–5.9 × 3.7–5.3 μm) with short warts (0.1–0.5 μm high).

Description. Unexpanded basidiomata epigeous, napiform to citriform, light brown (5D4), 4.3–8.0 mm × 4.0–7.4 mm, surface densely tomentose to rugulose when mature, not encrusted with debris. Subiculum white orange (5A2) to yellowish (4A2), covering the substrate, rhizomorphs light brown (5D4) attached to the substrate. Expanded basidiomata saccate, 2.9–7 mm high × 7.0–9.5 mm wide. Exoperidium splitting into 5–6 revolute, triangular rays, 3–4 mm diam, non–hygroscopic. Mycelial layer light brown (5D4) to orange white (N_00_Y_30_M_10_), surface papery, slightly tomentose to rugose, not encrusted with debris, persistent. Fibrous layer papery, yellowish white (4A2). Pseudoparenchymatous layer pale red (7A3) when fresh and light brown (6D4) to brown (N_70_Y_90_M_50)_ when dried, persistent or cracking on the base of rays. Endoperidial body brownish gray (6D2) to greyish brown (N_70_Y_40_M_20_), globose to subglobose, 3.0–5.5 × 3.0–6.0 mm sessile, glabrous. Peristome fibrillose, delimited by a line, brownish gray (6E2), lighter than endoperidium, mammiform, 2 mm wide, up to 1 mm high, slightly depressed on endoperidium. Columella cylindrical, 4.2 × 0.4 mm, central, conspicuous, yellowish white (3A2). Mature gleba brown (6E4).

Subiculum composed of hyaline, filamentous, slender hyphae, 0.9–1.2 μm diam, dextrinoid, sinuous. Rhizomorphs composed of hyaline, slender hyphae, lumen not evident, surface covered by crystals, with coarser and more irregular oblique prism shape, 1.1–2.9 × 6.2–8.2 μm. Mycelial layer formed of hyaline to yellowish hyphae, thin walled (1 < μm diam), 2.8–4.8 μm diam, dextrinoid, surface encrusted, lumen evident, unbranched. Fibrous layer formed of hyaline hyphae, 4.6–8.7 μm diam, surface not encrusted, lumen not evident. Pseudoparenchymatous layer formed by brownish hyphal cells, subglobose to pyriform, 29.4–53.2 × 22.5–40.5 μm, thin–walled (<1 μm). Eucapillitium 2.2–4.4 μm diam, thick–walled (>1 μm diam), sinuous, branched, surface slightly verrucose, encrusted, lumen evident, dark brown in 5% KOH. Basidia subglobose to clavate, 4.5–21.4 × 3–6.6 μm, 3–5 sterigmata, yellowish brown in 5% KOH. Basidiospores subglobose to oval, 3.8–5.9 × 3.7–5.3 μm (x = 4.3 ± 0.5 × 4.6 ± 0.4, Q_m_ = 1.05, n = 60), yellowish, ornamentation inconspicuous under LM, verrucose under SEM, warts short (0.1–0.5 μm high), slightly columnar, with planar to rounded tips, apiculus reduced surrounded by columnar processes.

Ecology and distribution. This species has a Neotropical distribution. Found in the biomes Tropical & Subtropical Moist Broadleaf Forests of Brazil (Pernambuco coastal forests ecoregion) and Martinique Island (Windward Islands moist forests), and in the biome Mangroves of Panamá (Southern Mesoamerican Pacific mangroves ecoregion) on decaying wood, fruiting is gregarious, rarely cespitose.

Additional material examined. Brazil, Paraíba, Areia, Mata do Pau Ferro, Trilha do Cumbe, 15 Jul. 2013, leg. J.O. Sousa, D.S. Alfredo & R.A Lima JM36 (UFRN–Fungos 2308, paratype, ITS sequence GenBank MH634994, LSU sequence GenBank MH635027). Panama, Coiba Island, 15 Nov. 1996, leg. F. Pando & M.P. Núñez (MA–Fungi 36141, paratype, ITS sequence GenBank KF988438, LSU sequence GenBank KF988568). Martinique, Le Robert, bois Pothau, 30 Aug. 2008, leg. C. Lécuru (LIP CL/MART 08.112, paratype, ITS sequence GenBank MH635007, LSU sequence GenBank MH635034).

Remarks. This species has small basidiomata (7–9 mm wide when expanded), exoperidium light brown, tomentose to rugulose when mature; pseudoparenchymatous layer reddish when fresh to light brown when mature; peristome slightly depressed on the endoperidium; basidiospores subglobose to oval (Q_m_ = 1.07) with 4.2–5.9 μm diam, warts short (0.1–0.5 μm high) with planar to rounded tips. Based on morphology, *G*. *rubropusillum* is very similar to *G*. *schweinitzii*, and this explains some misidentifications, such as the collection MA–Fungi 36141 (seqs. KF988438/KF988568), which was previously identified as *G*. *schweinitzii* [10] but it belongs to the species *G*. *rubropusillum*. According to our data, *G*. *schweinitzii* differs from *G*. *rubropusillum* by having lighter pseudoparenchymatous layers (whitish when fresh), peristome non–depressed on the endoperidium, and globose basidiospores. Another species similar to *G*. *rubropusillum* is *G*. *pusillipilosum*, which is distinguished by its densely hairy exoperidium and globose basidiospores (Q_m_ = 1.00).

## Discussion

This study uncovered a hidden richness of subiculose neotropical *Geastrum* species. From our analyses 12 species were recovered, mainly collected in South and Central America, of which six were species unknown to science. Thus, we confirm the underestimated biodiversity of the genus *Geastrum* in the Neotropical region, illustrated by two cases of species complexes involving *G*. *schweinitzii* and *G*. *hirsutum*.

The barcode sequence (ITS) of the isotype of *G*. *schweinitzii* (K (M) 180187) grouped with only one sequence (INPA 143435) in Clade 1. It is interesting to note that these two collections are from the same biogeomorphological region: the Guiana Shield, a pristine Amazonian area with minor modifications during landscape evolution [42] revealing a possible scenario for allopatric or parapatric speciation dynamics in which *G*. *schweinitzii* may be endemic, since the Pakaraima Mountains are an ecological barrier for many organisms [44,48,49]. Keeping this in mind, the ten synonyms of *Geastrum schweinitzii* proposed by Ponce de León [27] need to be reassessed. Furthermore, some of the putative synonyms are from very distinct ecoregions [5]: e.g. *G*. *papyraceus* Berk. & M.A. Curt. from Bonin Islands (Indomalayan Realm); *G*. *lignicola* Berk. from Australia (Australasia Realm); *G*. *rhizophorum* Dissing & M. Lange from Congo (Afrotropic Realm); and *G*. *subiculosum* from Australia (Australiasia Realm).

Fifteen samples with the morphology traditionally associated to the name *G*. *schweinitzii*, collected from widespread areas of the neotropical region, appear in clade XII in Fig 1, illustrating a case of semi-cryptic species [17,18,20,21] and evolutionary convergence in their morphology. Another case of semi-cryptic species of *G*. *schweinitzii* is *G*. *baculicrystallum*, the two species could be distinguished only by details in basidiospore size and ornamentation.

Three ITS sequences from GenBank previously identified under the name *G*. *schweinitzii*, KF988437, KF988438, and KF988439, are in fact three different species: *G*. *courtecuissei*, *G*. *rubropusillum*, and *G*. *pusillipilosum*, respectively. In these cases, morphological features distinguish each of these species.

The presence of hairs on the exoperidium is a recurrent feature of subiculose species. However, presence or absence of hairs as a single decisive feature for taxonomic identification could result in misidentification. Five of the species recognized here included this same feature. These semi-cryptic species are distinguished by molecular data, but discriminatory morphological features are unremarkable. *Geastrum brunneocapillatum*, *G*. *rubellum* and *G*. *hirsutum* are semi-cryptic species, and are not even sibling/sister species [17,18,20], reinforcing the statement that the presence of hairs on the exoperidium alone is not a suitable feature for species delimitation in *Geastrum*, but, instead, it represents an evolutionary convergence.

Recently a synonymization of *Geastrum trichiferum* to *G*. *hirsutum* was proposed [28]. *Geastrum trichiferum* is a mysterious species involved in taxonomic and nomenclatural problems in recent years [28,29,30]. Trying to better understand the nomenclature and taxonomic *status* of this species, we analyzed the collections PACA 15970 (packet labeled holotype in PACA), BPI 706088 (Rick’s original collection alleged by Zamora & Parra [30] and BPI 706086 (lectotype designated by Trierveiler-Pereira & Silveira [28] (S7 Fig).

It was possible to distinguish BPI and PACA collections from other species studied in this paper. By morphological analysis, we realized that these two exsiccates are notably different from each other, and they should probably be treated as distinct species: PACA 15970 has a basidiome with non-delimited peristome and small basidiospores (2.7–4 μm diam); while, BPI 706086 has delimited-peristome and larger basidiospores (4.4–6.5 μm diam). Thus, besides the nomenclatural problems involving its protologue, *G*. *trichiferum* has ambiguous type collections, since no voucher was indicated in the original description.

When compared to *G*. *hirsutum*, the collection BPI 706086 of *G*. *trichiferum* shows that specimens have larger basidiospores (4.4–6.5 μm diam) and lighter hairs (light brown), while morphologic analysis of the collection PACA 15970 shows that the single basidioma is distinct from *G*. *hirsutum* by the non–delimited peristome and short hairs on the exoperidium.

The type collections of *G*. *trichiferum* (S7 Fig) were also compared to *G*. *pusillipilosum*, a morphologically similar species, which also exhibits a hairy exoperidium. Our morphological analysis demonstrated that they can be differentiated mainly by basidiospore size and ornamentation. The collection PACA 15970 is distinguished from *G*. *pusillipilosum* by its non-delimited peristome and smaller basidiospores (2.7–4 μm diam) with inconspicuous ornamentation under LM, composed of small (0.1–0.6 μm high) warts with rounded tips under SEM; while the specimens of the BPI 706086 collection grow on a developed subiculum, the hairs on the exoperidium are longer (0.8–1.3 mm high), basidiospores have similar ornamentation: inconspicuous under LM, small (0.2–0.6 μm high) warts with rounded wart tips under SEM. Unfortunately, DNA extraction was not allowed by the herbarium and definitive conclusions are not possible.

We note that it is necessary to be extremely careful with species synonymization and consequent under-estimating of biodiversity. The integration of molecular and bioinformatic approaches for taxonomic and systematic studies seems to be essential for species delimitations in *Geastrum*, especially when dealing with species complexes.

## Supporting information

S1 Fig Maximum parsimony tree of ITS nrDNA sequences of *Geastrum* species. One sequence of *Geastrum velutinum* was used as out-group. Terminal branches are labeled with appropriate specimen codes. For further specimen details, see Table 1. Numbers at the nodes indicate the maximum parsimony bootstrap.
(TIF)

S2 Fig Maximum likelihood tree of ITS nrDNA sequences of *Geastrum* species. One sequence of *Geastrum velutinum* was used as out-group. Terminal branches are labeled with appropriate specimen codes. For further specimen details, see Table 1. Numbers at the nodes indicate the maximum likelihood bootstrap.
(TIF)

S3 Fig Maximum parsimony tree of concatenated ITS/LSU nrDNA sequences of *Geastrum* species. One sequence of *Geastrum velutinum* was used as out-group. Terminal branches are labeled with appropriate specimen codes. For further specimen details, see Table 1. Numbers at the nodes indicate maximum parsimony bootstrap.
(TIF)

S4 Fig Maximum likelihood tree of concatenated ITS/LSU nrDNA sequences of *Geastrum* species. One sequence of *Geastrum velutinum* was used as out-group. Terminal branches are labeled with appropriate specimen codes. For further specimen details, see Table 1. Numbers at the nodes indicate maximum likelihood bootstrap.
(TIF)

S5 Fig Bayesian tree of concatenated ITS/LSU nrDNA sequences of *Geastrum* species. One sequence of *Geastrum velutinum* was used as out-group. Terminal branches are labeled with appropriate specimen codes. For further specimen details, see Table 1. Numbers at the nodes indicate the posterior probabilities.
(TIF)

S6 Fig Geastrum mirabile. (A) Original collection of *G*. *mirabile* (PC0084351). (B, C) Japanese collection (TNS 36748) identified as *G*. *mirabile* by Kasuya et al. [16], adapted from http://db.kahaku.go.jp/webmuseum_en/mediaDetail?cls=col_b2_01&pkey=36748&lCls=med_b2_01&lPkey=B07-019855&detaillnkIdx=0. A, B, C bar = 10mm.
(TIF)

S7 Fig Collections of *Geastrum trichiferum*. (A) protologue in Lloyd (1907). (B) BPI 706088, material that correspond to Lloyd´s illustration (Fig 147–148), according to Zamora & Parra (2016), adapted from: http://nedoko.sakura.ne.jp/sblo_files/nedoko/image/RIMG2757.JPG (photo by Taiga Kasuya). (C) BPI 706086. (D) PACA 15970. (C1, D1) Herbarium data. (C2, D2) Basidiomata of collection. (C2, D3) Basidiospores under scanning electronic microscope. Bar C2, D2 = 5 mm, C3, D3 = 1 μm.
(TIF)
