# Supplementary figures and images for: Hidden fungal diversity from the Neotropics: Geastrum hirsutum, G. schweinitzii (Basidiomycota, Geastrales) and their allies
Source: PLoS One. 2019 Feb 6;14(2):e0211388. doi: 10.1371/journal.pone.0211388 (PMC6364924; doi:10.1371/journal.pone.0211388)

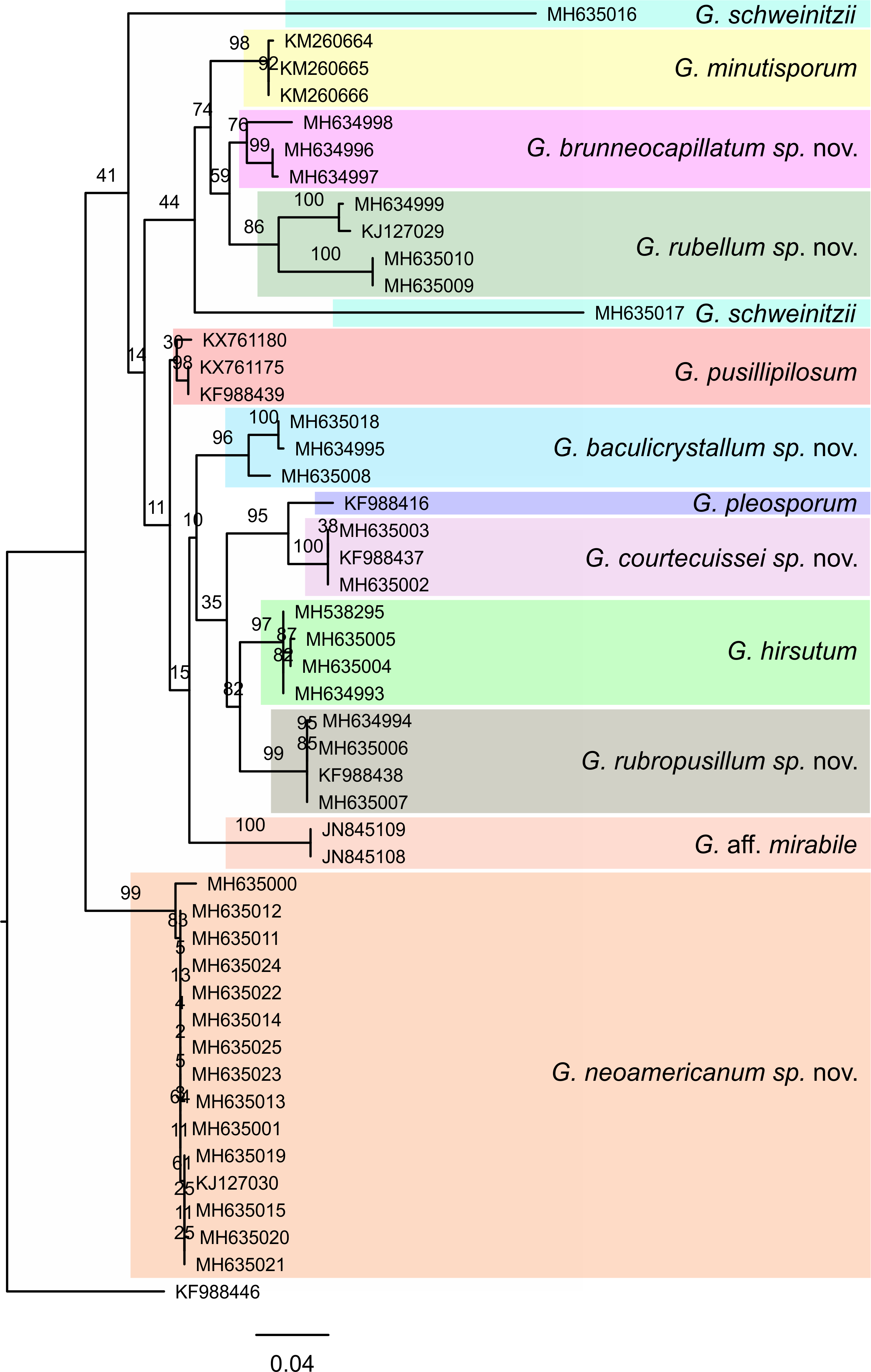

Supplement: S1 Fig — One sequence of Geastrum velutinum was used as out-group. Terminal branches are labeled with appropriate specimen codes. For further specimen details, see Table 1. Numbers at the nodes indicate the maximum parsimony bootstrap. (TIF) [file pone.0211388.s001.tif]

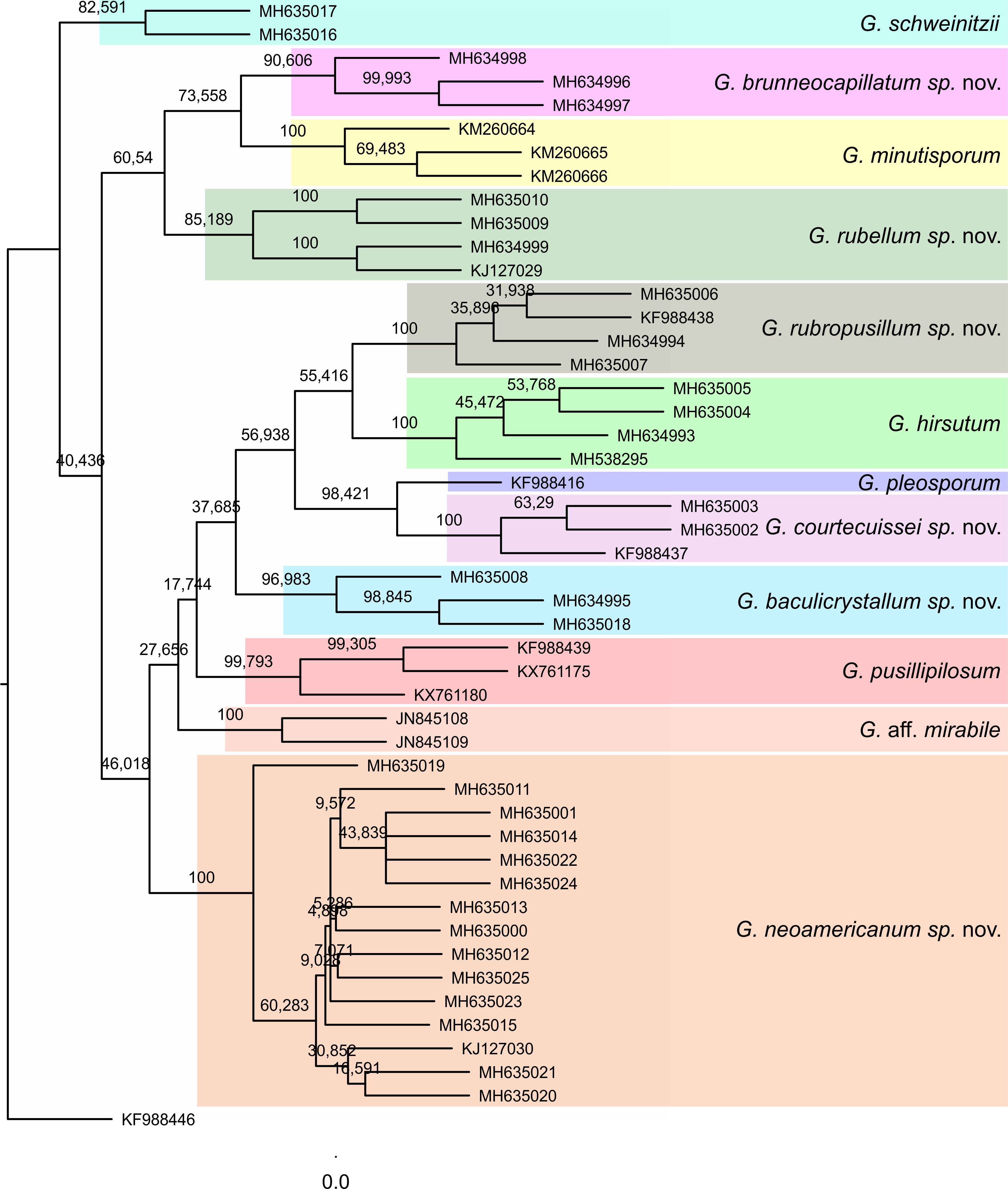

Supplement: S2 Fig — One sequence of Geastrum velutinum was used as out-group. Terminal branches are labeled with appropriate specimen codes. For further specimen details, see Table 1. Numbers at the nodes indicate the maximum likelihood bootstrap. (TIF) [file pone.0211388.s002.tif]

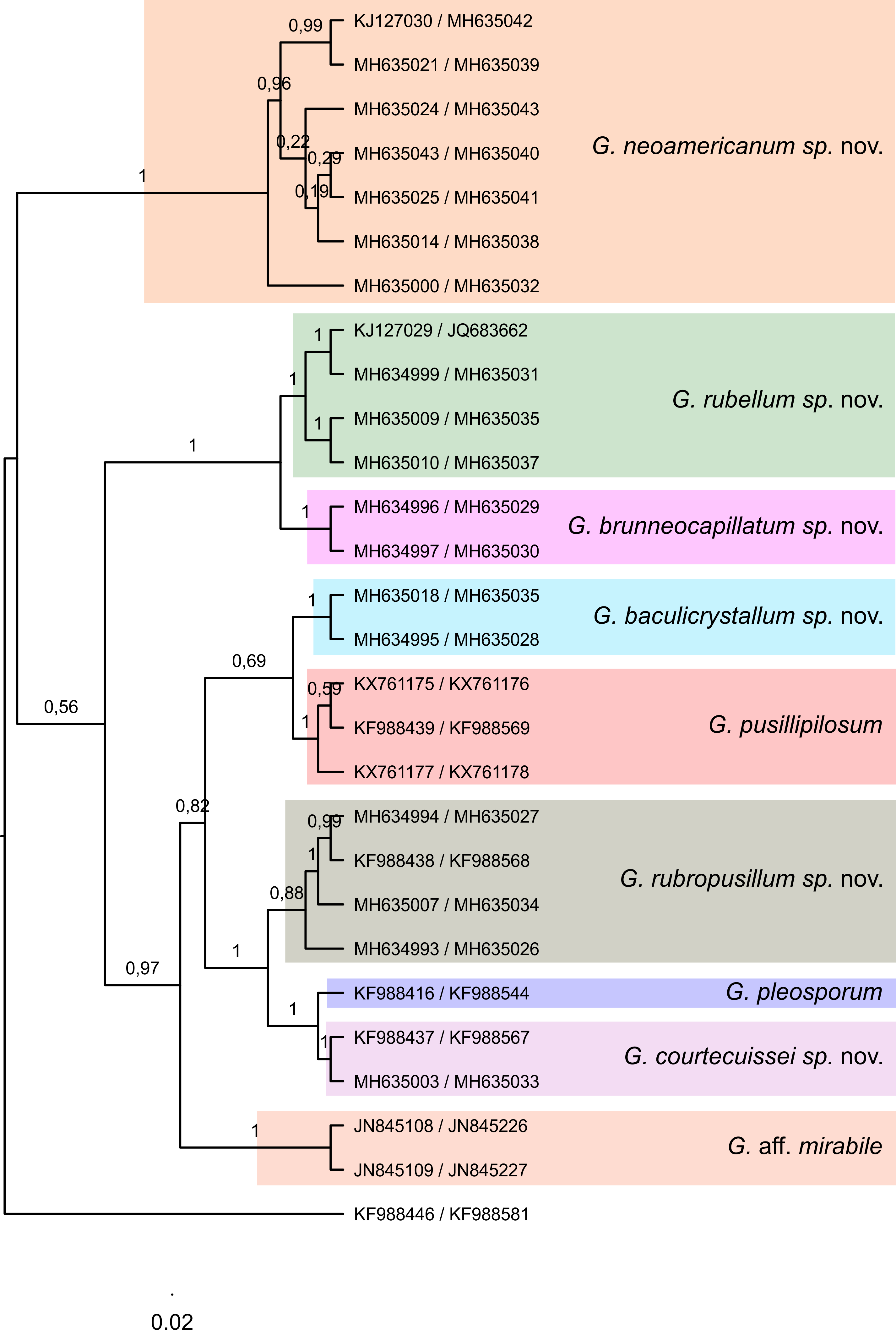

Supplement: S3 Fig — One sequence of Geastrum velutinum was used as out-group. Terminal branches are labeled with appropriate specimen codes. For further specimen details, see Table 1. Numbers at the nodes indicate maximum parsimony bootstrap. (TIF) [file pone.0211388.s003.tif]

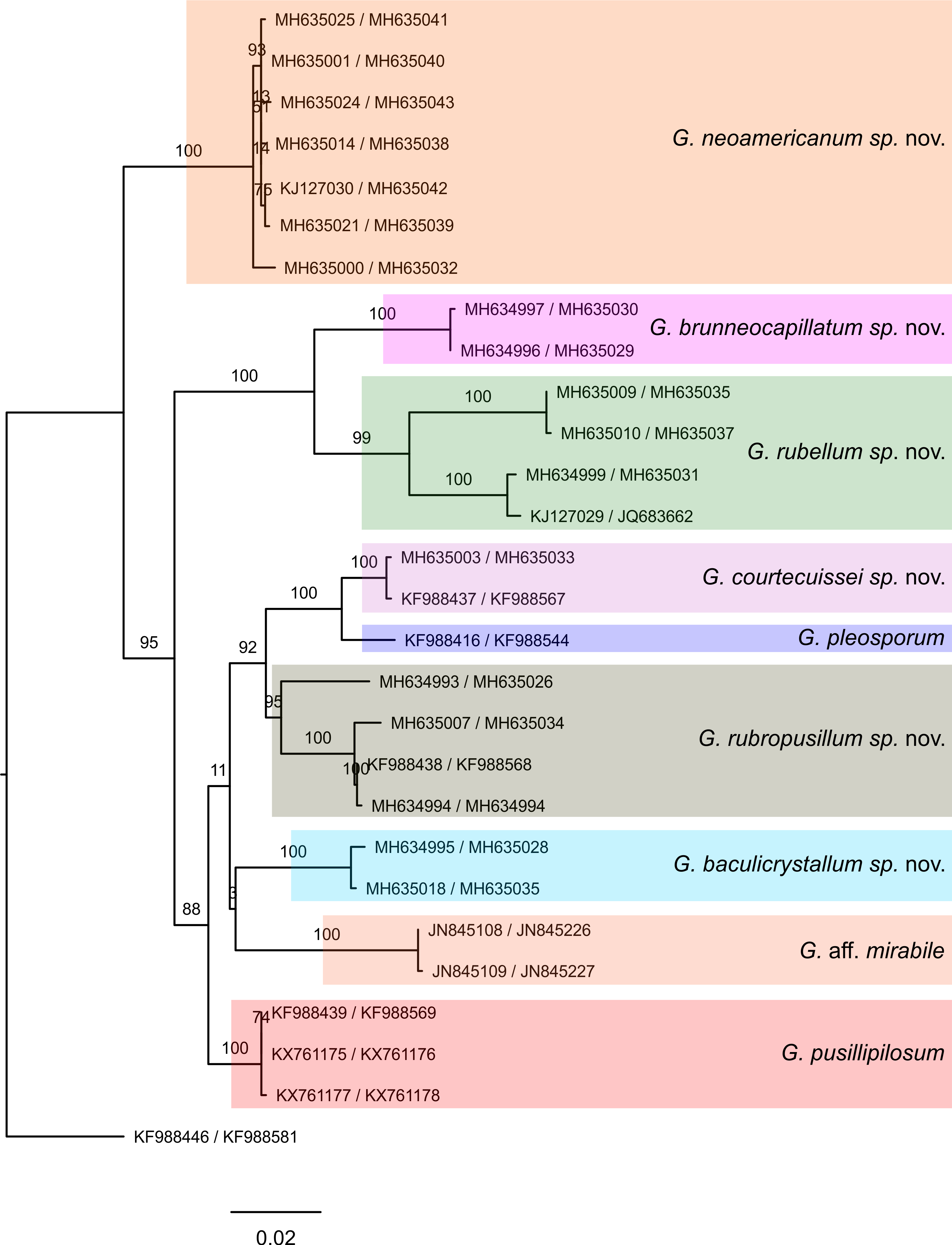

Supplement: S4 Fig — One sequence of Geastrum velutinum was used as out-group. Terminal branches are labeled with appropriate specimen codes. For further specimen details, see Table 1. Numbers at the nodes indicate maximum likelihood bootstrap. (TIF) [file pone.0211388.s004.tif]

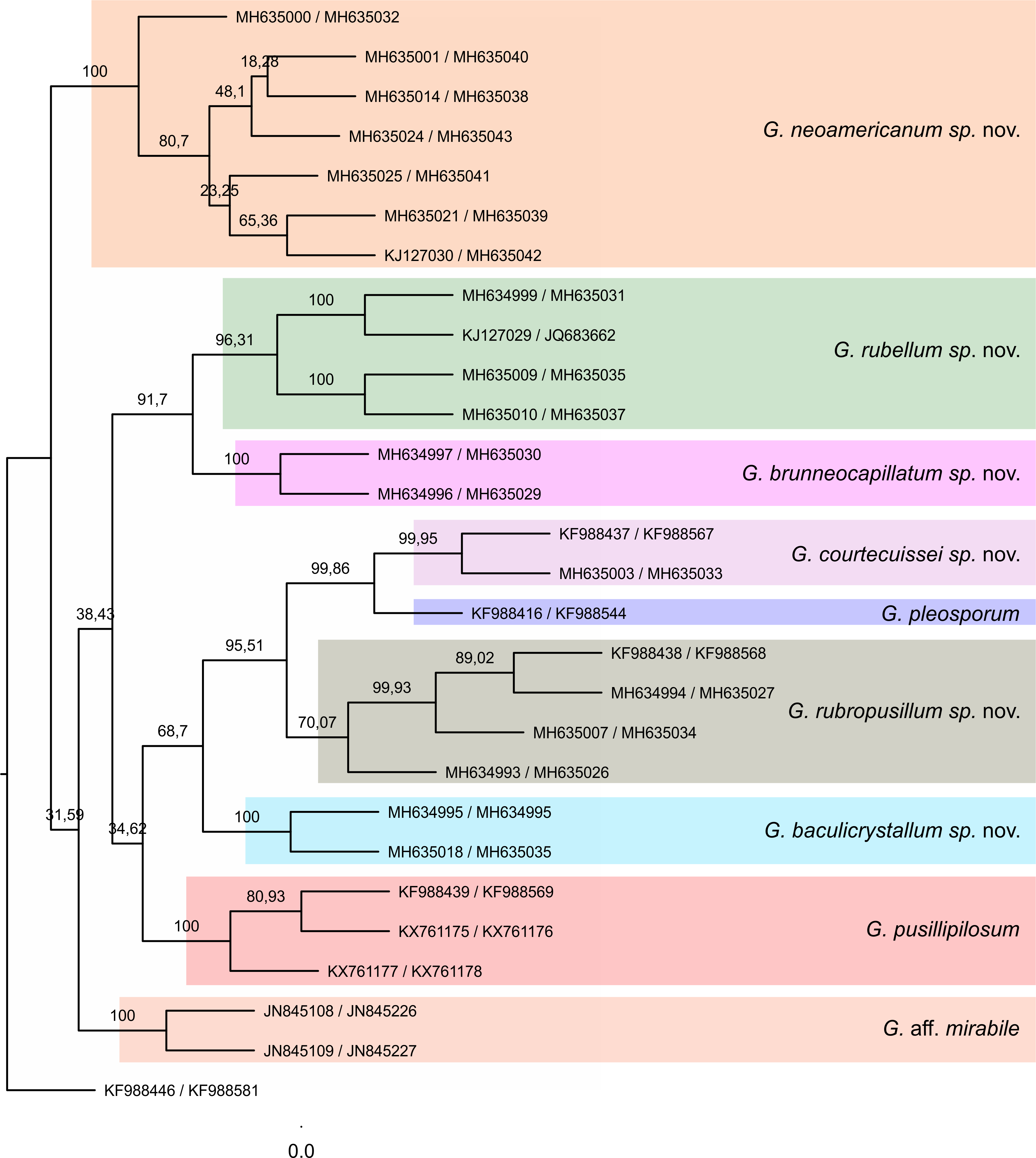

Supplement: S5 Fig — One sequence of Geastrum velutinum was used as out-group. Terminal branches are labeled with appropriate specimen codes. For further specimen details, see Table 1. Numbers at the nodes indicate the posterior probabilities. (TIF) [file pone.0211388.s005.tif]

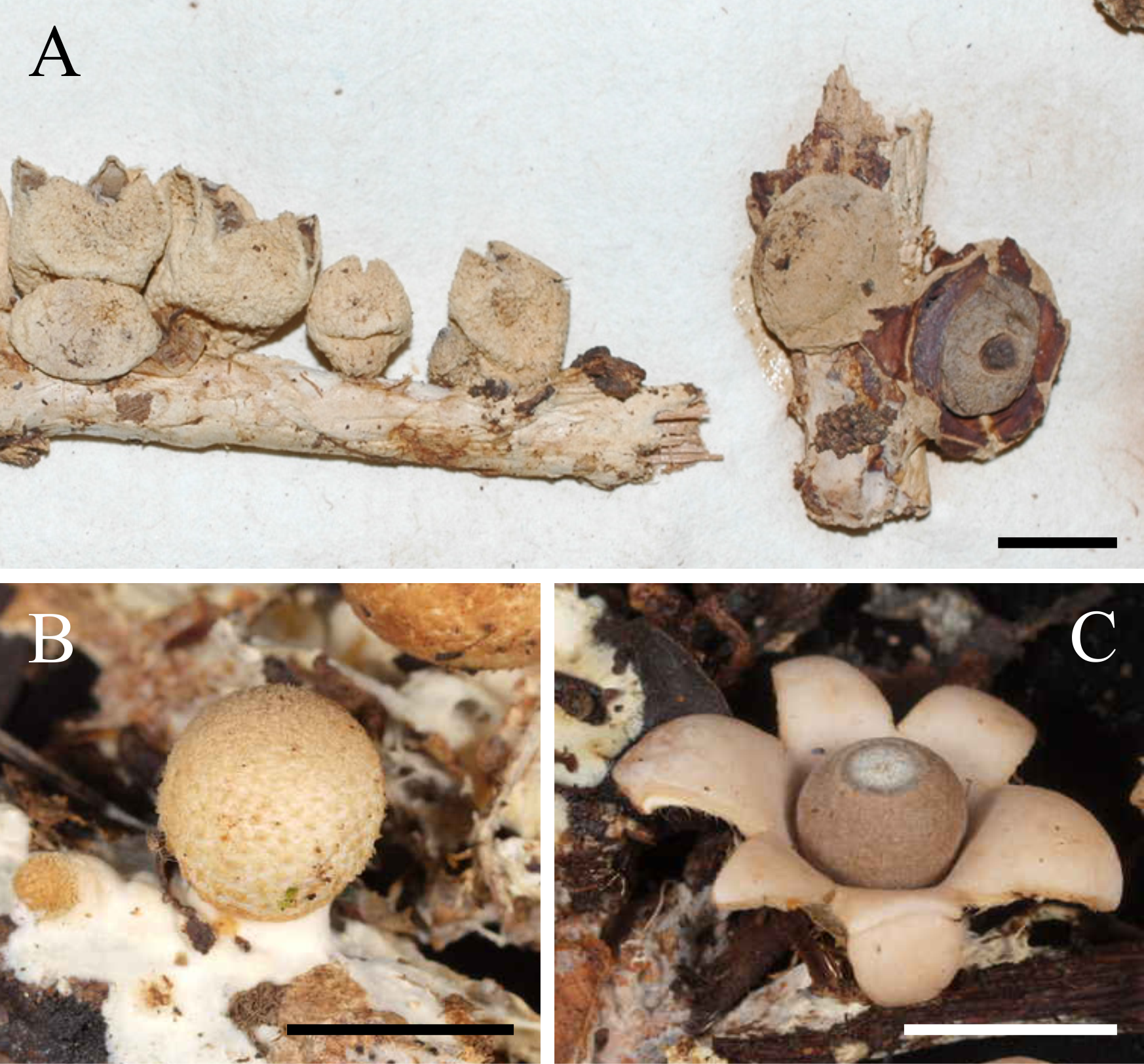

Supplement: S6 Fig — (A) Original collection of G. mirabile (PC0084351). (B, C) Japanese collection (TNS 36748) identified as G. mirabile by Kasuya et al. [16], adapted from http://db.kahaku.go.jp/webmuseum_en/mediaDetail?cls=col_b2_01&pkey=36748&lCls=med_b2_01&lPkey=B07-019855&detaillnkIdx=0. A, B, C bar = 10mm. (TIF) [file pone.0211388.s006.tif]

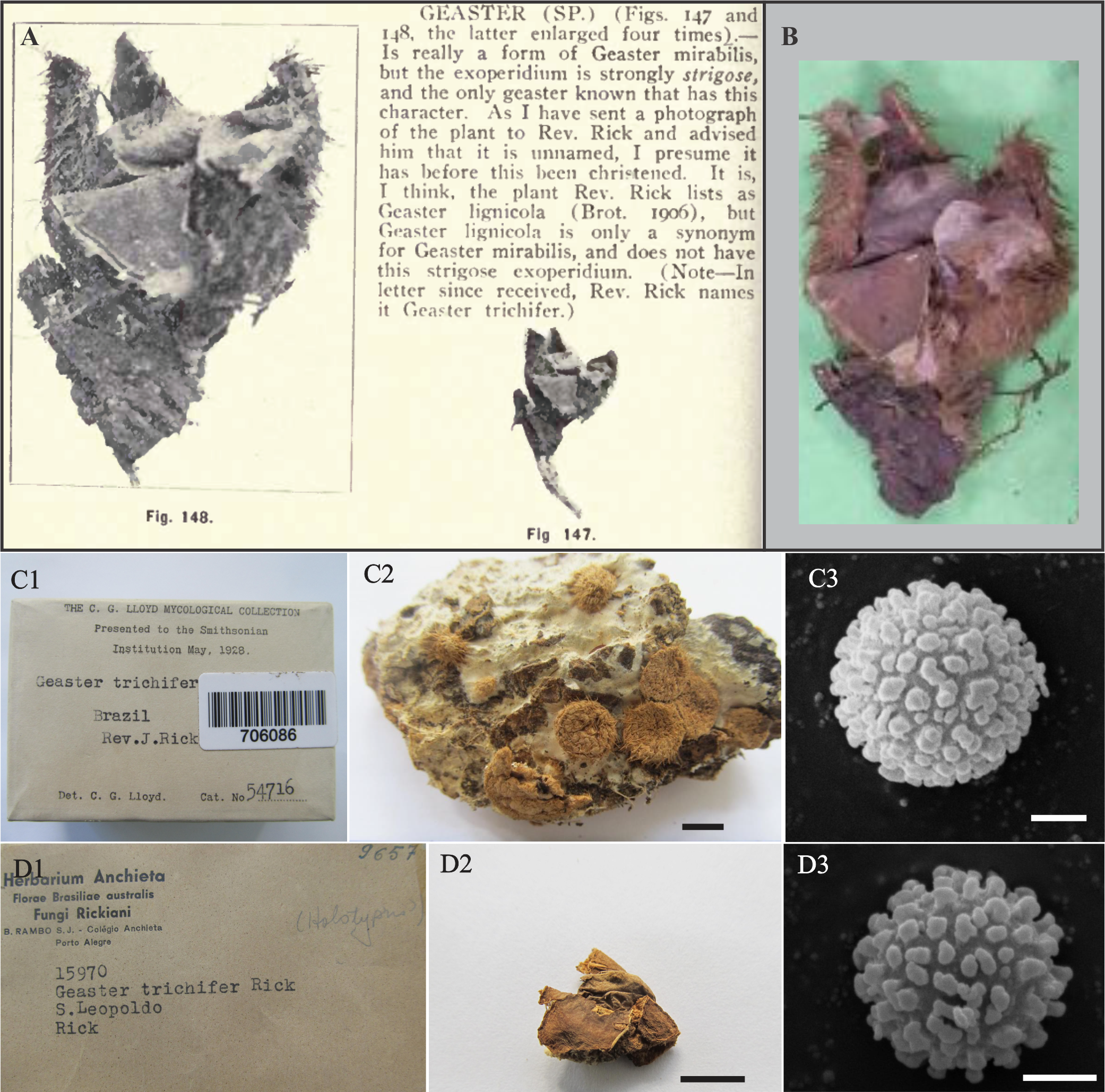

Supplement: S7 Fig — (A) protologue in Lloyd (1907). (B) BPI 706088, material that correspond to Lloyd´s illustration (Fig 147–148), according to Zamora & Parra (2016), adapted from: http://nedoko.sakura.ne.jp/sblo_files/nedoko/image/RIMG2757.JPG (photo by Taiga Kasuya). (C) BPI 706086. (D) PACA 15970. (C1, D1) Herbarium data. (C2, D2) Basidiomata of collection. (C2, D3) Basidiospores under scanning electronic microscope. Bar C2, D2 = 5 mm, C3, D3 = 1 μm. (TIF) [file pone.0211388.s007.tif]
